# Prevalence of nicotine, alcohol, and cannabis use disorders among adults with chronic pain: Protocol for a systematic review and meta-analysis

**DOI:** 10.1371/journal.pone.0356365

**Published:** 2026-08-26

**Authors:** Andrew H. Rogers, Emily L. Zale, Jessica M. Powers, Yohannes W. Woldeamanuel, Nell Aronoff, Laurel Mueller, Chung Jung Mun

**Affiliations:** 1 Division of Behavioral Medicine, Department of Medicine, Jacobs School of Medicine and Biomedical Sciences, University at Buffalo, SUNY, Buffalo, New York, United States of America; 2 Department of Pediatric Oncology, Roswell Park Comprehensive Cancer Center, Buffalo, New York, United States of America; 3 Department of Psychology, Binghamton University, SUNY, Binghamton, New York, United States of America; 4 Department of Psychology, University of Kansas, Lawrence, Kansas, United States of America; 5 Cofrin Logan Center for Addiction Research and Treatment, University of Kansas, Lawrence, Kansas, United States of America; 6 Division of Headache, Department of Neurology, Mayo Clinic Arizona, Phoenix, Arizona, United States of America; 7 University Libraries, University at Buffalo, Buffalo, New York, United States of America; 8 Edson College of Nursing and Health Innovation, Arizona State University, Phoenix, Arizona, United States of America; 9 Department of Psychiatry and Behavioral Sciences, Johns Hopkins School of Medicine, Baltimore, Maryland, United States of America; Universita Cattolica del Sacro Cuore Sede di Roma, ITALY

## Abstract

Chronic pain is a significant public health problem associated with substantial medical expenditures, decreased quality of life, and physical and mental health comorbidities. Substance use is an important clinical consideration in pain, as substance use has been identified as both an antecedent and consequence of chronic pain. The majority of research on substance use and chronic pain has focused on opioid use, and although commonly used, alcohol, nicotine, and cannabis have received considerably less research attention. While there are empirical studies that examine the impact of non-opioid substance use in the context of chronic pain, the overall prevalence rates of nicotine, alcohol, and cannabis use disorders among adults with chronic pain have not been well-documented. Therefore, a systematic review and meta-analysis that characterizes the prevalence rates of non-opioid substance use disorders among adults with chronic pain is urgently needed. The current paper outlines the protocol for a systematic review and meta-analysis. It will include studies of adults with chronic pain that report on nicotine, alcohol, or cannabis use disorders, as well as problematic use (including co-use where available). The following databases will be searched: PubMed, Embase (Elsevier), Web of Science Core Collection, CINAHL (EBSCO), and PsycINFO (EBSCO). A team of trained research staff will conduct study screening and data extraction. Analyses will focus on pooled prevalence estimates of substance use disorders as primary outcomes and problematic use as secondary outcomes, with planned sub-group analyses and strategies to mitigate potential sources of bias. The results of this review will provide valuable clinical information on the scope of non-opioid substance use disorders, helping to guide future research as well as inform clinical and policy-level decision making.

## Introduction

Chronic pain, defined as pain that has been present for ≥3 months [1], negatively impacts a significant proportion of the population worldwide [2–4]. The estimated prevalence of chronic pain differs based on country, definition, and assessment method. Yet, primary and meta-analytic research suggests that between one-fifth and one-third of adults worldwide experience chronic pain [5–8]. These often include chronic overlapping pain conditions, a group of frequently co-occurring pain conditions that include chronic low back pain, irritable bowel syndrome, chronic pelvic pain, fibromyalgia, and migraine, among others [9–12]. Chronic pain is associated with significant medical, psychiatric, and economic costs of upwards of $1 trillion a year [13,14]. The impact of chronic pain extends beyond economic and healthcare, negatively impacting quality of life [15]. Chronic pain is associated with increased rates of mental health symptoms and disorders [16,17], as well as substance use disorders [18,19].

Substance use in the context of chronic pain is of particular clinical importance given pain has been identified as a potent motivator for substance use [20,21]. Substance use often serves a regulatory function for managing multiple facets of pain [22], amplifying negative pain-related outcomes, and contributing to adverse health consequences [19]. Further, substance use disorders (SUDs) have been documented in higher prevalence rates among individuals with chronic pain than those without, conferring risk for poorly managed pain and problematic substance use [23–26]. To date, most attention has been focused on opioids, where prescriptions for pain have been regarded as primary drivers of persistent opioid use, the opioid epidemic, and related consequences [27,28]. Meta-analytic evidence provides support for overall higher rates of opioid use disorder among adults with chronic pain than those without [29–32]. Similarly, nearly half of all individuals receiving treatment for opioid use disorder also meet criteria for chronic pain [33–35], which further hinders opioid use disorder recovery processes [36]. Emerging evidence indicates that most widely used non-opioid substances, specifically nicotine, alcohol, and cannabis use, are frequently used for the self-management of chronic pain. In addition, these substances are often used *with* opioids, and their chronic or problematic use (i.e., use that engenders increased risks for negative physiological or psychosocial consequences) is similarly linked to negative health outcomes and decreased quality of life [29,37]. Challenges in accurately capturing the extent to which individuals with chronic pain have SUDs involving these non-opioid substances have limited the empirical literature in this area, contributing to the wide variability in estimates of prevalence rates.

Theoretical models of chronic pain and substance use posit a bidirectional association in which pain motivates substance use, while chronic substance use exacerbates pain through several biopsychosocial mechanisms [38–42]. Adults with chronic pain are more likely to use nicotine, alcohol, and cannabis compared to those without chronic pain [43–45], and similarly, high rates of chronic pain are reported among individuals who use these substances [43,44,46,47]. Despite these empirical data, to date, the overall prevalence of nicotine, alcohol, and cannabis use disorders among adults with chronic pain has not been well quantified. A systematic review and meta-analysis published in 2011 found that between 3% and 48% of adults with chronic pain met criteria for any SUD, including opioid use disorder [23]. However, not only was it conducted more than 10 years ago, but the meta-analysis also excluded studies of nicotine use disorder, and the searches could have been more comprehensive.

The current state of knowledge regarding the prevalence rates of nicotine, alcohol, and cannabis use disorders, along with problematic use, among adults with chronic pain is limited in scope and imprecise. There is urgent need for clearer understanding of these prevalence rates, as they can inform the development of both treatment and prevention programs and policy efforts addressing the intersection of chronic pain and non-opioid SUDs. It is also important to quantify the rates of problematic use, as even use that does not meet diagnostic criteria for a SUD can have long term physiological and psychosocial consequences [48]. Accordingly, the proposed systematic review and meta-analysis aims to fill this gap by consolidating existing knowledge and providing estimates of nicotine, alcohol, and cannabis use disorders (primary outcome), as well as estimates of problematic substance use (secondary outcome), among adults chronic pain conditions.

## Materials and methods

The protocol was guided by the Preferred Reporting Items for Systematic Review and Meta-Analyses for Protocols (PRISMA-P) checklist (see appendix for checklist; 49). The protocol was pre-registered with the International Prospective Register of Systematic Reviews (PROSPERO) on November 10, 2024 (updated on July 24, 2026 prior to the initiation of the search and data extraction), under registration number CRD42024596125. Currently, the search strategy has been generated and piloted, but the full searches have not been conducted. It is expected that, following the searches, data extraction and results will be available within 12 months.

### Eligibility criteria

For full inclusion and exclusion criteria, see S1 Table. Primary study outcomes will focus on nicotine, alcohol, and cannabis use disorders, and secondary outcomes will focus on problematic use of these substances (see S2 Table for definitions). Studies that are focused on exclusively cancer-related pain will be excluded.

### Information sources

The following types of studies will be included: original/empirical data, such as cross-sectional studies, cohort studies, and both non-randomized and randomized controlled trials. For longitudinal and interventional studies, only baseline assessments prior to randomization/intervention exposure will be used, rather than mid-treatment, post-treatment or incidence data. The following types of studies will be excluded: literature reviews, systematic reviews, case reports, protocols, commentaries, conference abstracts, opinions, and letters. Additionally, studies that specifically recruit participants based on SUD criteria will be excluded to avoid inflated prevalence estimates.

### Search strategy

The following five databases will be searched for potential studies: PubMed, Embase (Elsevier), Web of Science Core Collection, CINAHL (EBSCO), and PsycINFO (EBSCO). The searches were designed by two librarians (NA and LM) to capture papers about adults that experience chronic pain conditions [11,12,49] and report problematic nicotine, alcohol, or cannabis use or use disorders. The searches have the following general structure: chronic pain AND ((substance AND misuse) OR substance misuse). They include broad terms like chronic pain and persistent pain, as well as specific chronic overlapping pain conditions [50]. Keywords and subject headings were employed to help ensure comprehensiveness. Six articles were used as seed articles [51–56]. Seed articles are those the research team anticipates including in the review, and the goal is to design searches that successfully retrieve these articles. One seed article did not come up in any of the searches because it did not contain any misuse-related terms, despite the inclusion of a comprehensive set of synonyms reflecting aspects of problematic use. Publication types will be restricted in Embase (article, article in press, clinical trial), Web of Science Core Collection (article or early access), CINAHL (academic journals), and PsycINFO (academic journals) in accordance with our inclusion and exclusion criteria. The full PubMed search strategy is in S1 Appendix. This search strategy serves as the template for the other four databases.

### Study selection/article screening

Study citations will be exported to Covidence. After duplicates have been removed in Covidence, titles and abstracts will be screened by two independent reviewers. Any disagreements on article inclusion will be resolved by a third reviewer. Once the potentially relevant articles are identified, full texts will be retrieved and compared to the full inclusion and exclusion criteria by two independent reviewers. For papers in languages other than English, the abstracts will be reviewed using machine translation tools (e.g., Google Translate) for usable data during the full-text screening stage. Due to restrictions in funding for formal translation services, articles that appear to meet inclusion criteria that the team is unable to translate will be excluded. For these articles, we will 1) indicate in the PRISMA flow diagram how many of these articles were identified, and 2) provide a summary table in the appendix that includes, when available from the abstract, the study country, pain condition, substance type, and reported prevalence (excluding non-English full texts may be a source of language and regional bias. If a clear pattern is identified from these non-English articles, we will note this in the main text.). A table of these articles will be included in the appendix. Discrepancies will be resolved by a third reviewer. Any studies that do not meet the inclusion criteria will be excluded, and exclusion reasons will be documented in a PRIMSA flow diagram according to PRISMA 2020 reporting guidelines [57]. If the searches are older than 12 months, they will be re-run.

### Data extraction and management

Once the list of included articles has been finalized, data will be extracted from each study by two independent coders. For each study, extracted data will include study details (author, year of publication), study characteristics (study location; sample size; sampling method; study design; study setting; demographics), pain characteristics (pain condition, intensity, duration, frequency), substance use characteristics (frequency and quantity of alcohol, nicotine, and/or cannabis use; prevalence rates of nicotine, alcohol, or cannabis use disorders; prevalence of problematic substance use, types of products for nicotine and cannabis; routes of administration for nicotine and cannabis; and method of assessment). Prevalence estimates for problematic substance use will only be derived from validated screening instruments with established cutoffs (e.g., AUDIT) and will also include the specific construct being assessed (e.g., hazardous use, misuse, etc.); studies that rely solely on author-defined thresholds for problematic substance use will not contribute to pooled prevalence estimates. Additionally, specific estimates of co-use (i.e., concurrent use disorders or problematic use involving multiple substances) will be extracted. Where sufficient data are available, co-use will also be examined in exploratory analyses; however, co-use estimates will not be included in the primary pooled prevalence analyses for individual substances.

### Risk of bias assessment

Utilizing the Joanna Briggs Institute (JBI)’s Critical Appraisal Checklist for Studies Reporting Prevalence Data, two independent reviewers will evaluate the methodological quality of each study and assessing the risk of bias [58]. Any disagreements between the reviewers will be resolved through discussion and, when necessary, consultation with a third reviewer. However, risk of bias will not be interpreted solely on the basis of an overall summary score. In additional to conducting sensitivity analyses excluding studies judged to be at high risk of bias, we will report the individual JBI domain rating for each study included in the supplementary materials. This approach will assist with interpretation of the findings. In addition, to investigate the presence of small study publication bias, we will use the funnel plot and contoured funnel plot for a visual inspection of study bias [59], and Egger’s regression test for a statistical test of small study bias [60]. When Egger’s test is significant, suggesting the presence of small study publication bias, we will employ Duval and Tweedie’s trim and fill procedure [61], which calculates how many studies are missing, imputes effect sizes from the missing studies, and estimates what the effect size would have been if the missing studies had been included.

### Data analysis and synthesis

Data analyses will be conducted using R to calculate the pooled prevalence point estimates (with 95% confidence intervals) of nicotine, alcohol, and cannabis use disorders (primary outcome) and problematic nicotine, alcohol, and cannabis use (secondary outcome) among individuals with chronic pain. To address assumptions of independence of each study, only one prevalence rate per substance will be included in the pooled analysis. When studies provide a total sample estimate, no additional subgroup prevalence rates will be extracted. If studies report multiple prevalence rates (e.g., subgroups) for the same substance, a single study-level prevalence estimate will be derived by aggregating subgroup data using sample size weighted estimates (i.e., total number of cases divided by the total sample size). This retains within-study information while minimizing bias associated with unweighted average of prevalence rates and ensures each study contributes only one independent estimate per substance [62]. This approach is in line with existing meta-analytic prevalence research [63–67]. We will also consider conducting sensitivity analyses using alternative strategies, including selecting the largest subgroup, as well as serially excluding each study to determine the influence of individual studies on the overall prevalence estimates. When studies report multiple thresholds (e.g., different severity cutoffs), we will prioritize estimates based on standard validated thresholds (e.g., AUDIT ≥8) or the threshold identified by the authors as primary. In terms of the time frame, where sufficient data are available, we will prioritize past year (or current) estimates for both SUD diagnoses and problematic use, given their greater clinical relevance and comparability across studies. Lifetime estimates will be analyzed separately and not pooled with past-year estimates.

Where sufficient data are available, co-use will also be examined in exploratory analyses; however, co-use estimates will not be included in the primary pooled prevalence analyses for individual substances. Because analyses focus on substance-specific prevalence, individuals who report multiple substances may contribute to more than one substance-specific analysis (e.g., both alcohol and nicotine). Importantly, individuals will not be counted more than once. We will descriptively report how co-use was defined in each of the estimates used. Further, in an effort to eliminate potentially duplicate study prevalence estimates, studies will be screened according to published recommendations [68], including examining the same first author, corresponding author, sample size, study year, study measures/prevalence rates, cohort or database name (when applicable), recruitment period, and other relevant study characteristics (e.g., geographic location). For studies that have been determined to be from the same sample and reporting the same prevalence outcome, only one independent estimate will be included in the meta-analysis, with preference given to the study with the largest eligible sample and/or the most comprehensive prevalence data relevant to the planned analysis.

Several sensitivity and moderator subgroup analyses will be examined based on available data. Specifically, point prevalence estimates will be examined as a function of study setting, pain characteristics, biological sex, study location, type of product (for nicotine and cannabis), route of administration (for nicotine and cannabis), medical vs. non-medical use and legal status (for cannabis where sufficient information is available), screening instrument for problematic use, study quality (utilizing the Joanna Briggs Institute Critical Appraisal Checklist for Studies Reporting Prevalence Data sum score), study type (e.g., cross-sectional, longitudinal), and age [58]. Additionally, we will include risk of bias scores in sensitivity analyses along with descriptive reporting.

For the meta-analysis, a random-effects model, using the restricted maximum likelihood (REML) estimator for between-study heterogeneity, will be used first to estimate the pooled prevalence rates for each outcome using generalized linear mixed models (GLMMs) with a binomial distribution and logit link [69,70]. Because this relies on a binomial distribution, variance-stabilizing transformations are not required [71]. To test the homogeneity assumption for meta-analyses, I² and τ^2^ will be examined. Based on prior research and recommendation, I² will not be interpreted using rigid thresholds, as very high I² values (e.g., > 90%) are common in prevalence meta-analyses and may not meaningfully distinguish heterogeneity [72]. Rather, I² will be interpreted in conjunction with τ^2^, prediction intervals, and the clinical and methodological comparability of included studies [72]. Quantitative synthesis will be undertaken only when studies are judged to be sufficiently comparable with respect to key conceptual characteristics, including the chronic pain population, substance definitions, measurement approach, and study designs [73–75]. If studies are judged to be too conceptually heterogeneous for a pooled prevalence estimate (determined by study team consensus), we will instead provide a narrative synthesis and visual presentation with the range of prevalence rates across studies. With substantial heterogeneity, we will interpret descriptive summaries rather than single estimates, emphasizing prediction intervals, subgroup patterns, and the limits of generalizability

**Fig 1:**
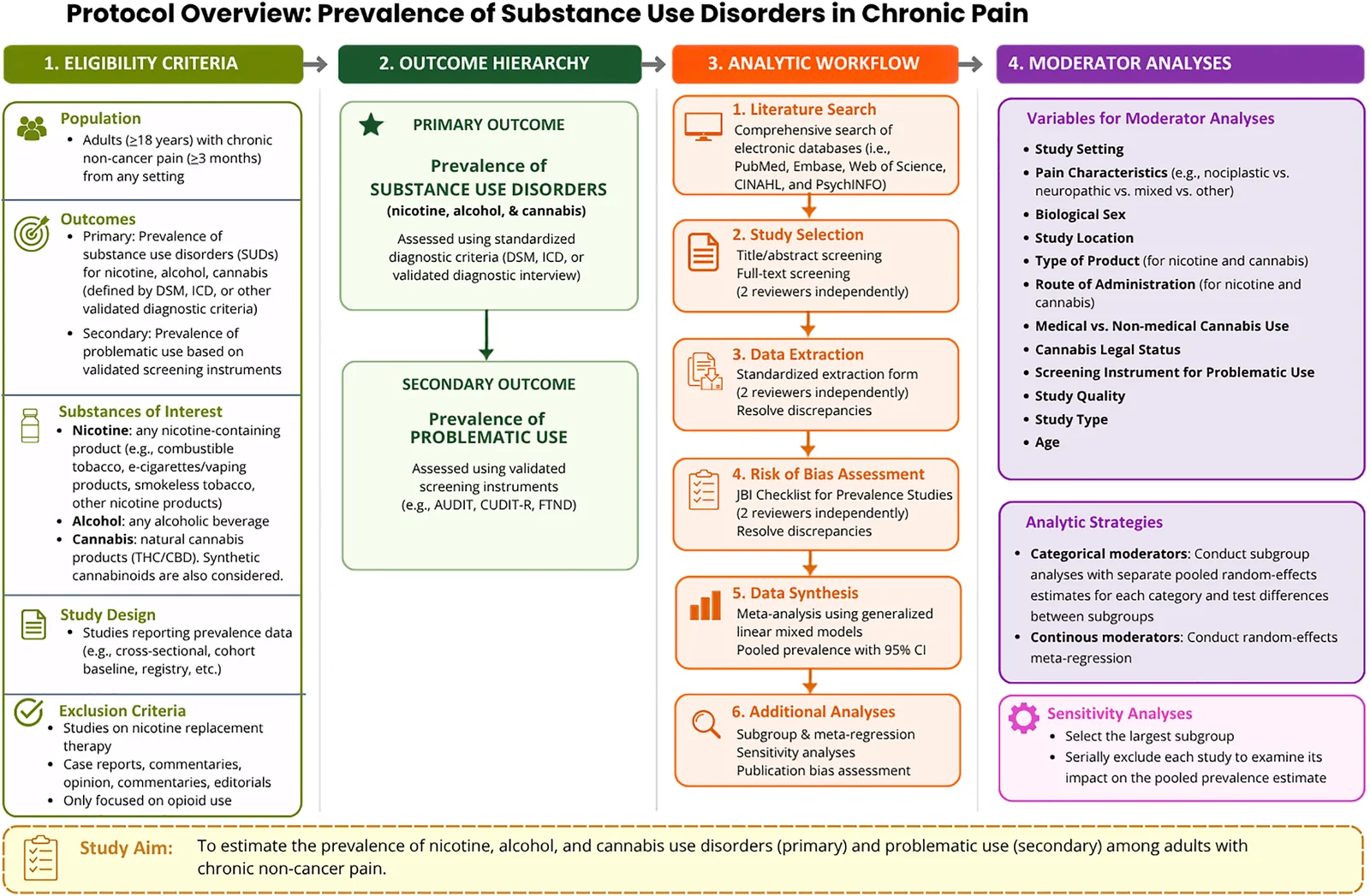
Protocol Overview: Prevalence of Substance Use Disorders in Chronic Pain. Flow chart for meta analysis, guiding through steps to determine eligibility criteria, outcomes, analytic workflow, and individual analyses. Moderator and sensitivity analyses represent currently identified moderators. It is possible there will be additional moderators identified upon completion of the search and data extraction.

To examine the effect of potential moderators on study heterogeneity, two types of moderator analyses will be conducted. First, for categorical moderators, sub-group analyses allowed for individual pooled random-effects correlations for each group studied, as well as a statistical test of between-study variability [65]. For continuous moderators, a meta-regression analysis will be conducted [76]. Pooled estimates will be presented using random-effects models even in the presence of substantial heterogeneity, but will be interpreted cautiously, as average prevalence across diverse contexts rather than precise summary values, including prediction intervals. If there is significant variability in the number of studies that include each moderator variable, not all studies will be included in each moderator analysis. While past research suggests that a minimum of two studies is needed to conduct a meta-analysis [77], we will adopt a more conservative threshold of five studies [78], given the random-effects model and the potential for significant within- and between-study heterogeneity. All subgroup and meta-regression analyses will be conducted based on *a priori* hypotheses to explore potential sources of heterogeneity. If heterogeneity is judged to be too substantial to support meaningful pooled estimates, we will supplement or replace pooled estimates with a narrative synthesis focused on the distribution and range of estimates across studies Fig 1.

### Ethics

Ethics approval is not required, as the study will rely on published data.

## Discussion

The proposed study will address an important gap by providing a clearer and more accurate picture of nicotine, alcohol, and cannabis use disorders, as well as problematic use among adults with chronic pain. Although non-opioid substance use in this population is quite common, the prevalence of each corresponding use disorder remains poorly understood. The lack of research on prevalence of SUDs in chronic pain can detrimentally impact clinical care if providers underestimate or overlook substance use in the context of chronic pain management, or lack sufficient understanding of the underlying mechanisms, treatment options, and preventative approaches. The goal of this meta-analysis is to provide much-needed data for patients, providers, and policy makers, and to guide future research inquiries, as well as development of intervention and prevention strategies for individuals with chronic pain and SUDs.

There are, however, several limitations to the proposed meta-analysis which are important to acknowledge. First, we plan to restrict the articles to peer-reviewed studies and do not have the resources to translate full length articles written in languages other than English. This may introduce potential bias into prevalence estimates, with potential underrepresentation of estimates from low- and middle-income countries, as well as not fully reflecting the global epidemiology of substance use disorders among individuals with chronic pain. Additionally, we expect that there will be substantial heterogeneity in how SUDs and problematic substance use are assessed and reported across studies, including variability in diagnostic criteria, screening measures, and chronic pain characteristics. These variabilities may limit our understanding of observed differences and interpretability of pooled findings. Although we do plan to conduct sub-group analyses to address these potential sources of heterogeneity, our ability to disentangle these factors is limited by how they are reported in existing literature. The expected heterogeneity may further impact the generalizability of the findings, and interpreting the prevalence estimates with caution will be warranted. The proposed meta-analysis has the potential to significantly advance the literature by providing more accurate understanding of the scope of nicotine, alcohol, and cannabis use disorders among adults with chronic pain. These findings can inform several actionable next steps for key stakeholders. For clinicians, the results may support the routine implementation of validated screening tools for SUDs in chronic pain treatment settings and the integration of brief interventions or referrals when necessary. For researchers, the findings may help identify some gaps in measurements and reporting practices, guiding the development of more standardized assessments. At the policy level, the results may inform resource allocation and the development of integrated care models that address co-occurring chronic pain and SUDs. Collectively, this meta-analysis will contribute to the development of more targeted and effective prevention and treatment for this potentially prevalent and a burdensome public health challenge.

## Supporting information

S1 Table Inclusion and Exclusion Criteria for Reviewed Studies. Mixed cancer-pain and non-cancer pain populations will be included only if specific data on non-cancer pain can be extracted from the article. Nicotine-related outcomes may include combustible tobacco, electronic cigarettes/vaping products, smokeless tobacco, and other nicotine-containing products when assessed as part of nicotine use disorder or problematic nicotine use. Cannabis-related outcomes may include synthetic cannabinoids.
(DOCX)

S2 Table Terminology referring to problematic substance use. The list of terms above identified possible terms that indicate problematic substance use [79–85]. Following the full article search and data extraction, and updated table will be included in the published manuscript.
(DOCX)

S1 File Inclusion and Exclusion Criteria for Reviewed Studies. (DOC)

S1 Appendix PubMed Search Strategy. (DOCX)

## References

[1] Treede R-D, Rief W, Barke A, Aziz Q, Bennett MI, Benoliel R, et al. Chronic pain as a symptom or a disease: the IASP Classification of Chronic Pain for the International Classification of Diseases (ICD-11). Pain. 2019;160(1):19–27. doi: 10.1097/j.pain.0000000000001384 30586067

[2] Jackson T, Thomas S, Stabile V, Han X, Shotwell M, McQueen K. Prevalence of chronic pain in low-income and middle-income countries: a systematic review and meta-analysis. The Lancet. 2015;385:S10. 10.1016/S0140-6736(15)60805-4 26313056

[3] Sá KN, Moreira L, Baptista AF, Yeng LT, Teixeira MJ, Galhardoni R, et al. Prevalence of chronic pain in developing countries: systematic review and meta-analysis. Pain Rep. 2019;4(6):e779. doi: 10.1097/PR9.0000000000000779 31984290 PMC6903356

[4] Kuehn B. Chronic pain prevalence. JAMA. 2018;320(16):1632. 10.1001/jama.2018.16009 30357307

[5] Fayaz A, Croft P, Langford RM, Donaldson LJ, Jones GT. Prevalence of chronic pain in the UK: a systematic review and meta-analysis of population studies. BMJ Open. 2016;6(6):e010364. doi: 10.1136/bmjopen-2015-010364 27324708 PMC4932255

[6] Murray CB, de la Vega R, Murphy LK, Kashikar-Zuck S, Palermo TM. The prevalence of chronic pain in young adults: a systematic review and meta-analysis. Pain. 2022;163(9):e972–84. doi: 10.1097/j.pain.0000000000002541 34817439

[7] Steingrímsdóttir ÓA, Landmark T, Macfarlane GJ, Nielsen CS. Defining chronic pain in epidemiological studies: a systematic review and meta-analysis. Pain. 2017;158(11):2092–107. doi: 10.1097/j.pain.0000000000001009 28767506

[8] Dahlhamer J, Lucas J, Zelaya C, Nahin R, Mackey S, DeBar L. Prevalence of chronic pain and high-impact chronic pain among adults - United States, 2016. MMWR Morb Mortal Wkly Rep. 2018;67(36):1001–6. 30212442 10.15585/mmwr.mm6736a2 PMC6146950

[9] Fitzcharles M-A, Cohen SP, Clauw DJ, Littlejohn G, Usui C, Häuser W. Nociplastic pain: towards an understanding of prevalent pain conditions. Lancet. 2021;397(10289):2098–110. doi: 10.1016/S0140-6736(21)00392-5 34062144

[10] Clauw DJ. Fibromyalgia: an overview. Am J Med. 2009;122(12 Suppl):S3–13. doi: 10.1016/j.amjmed.2009.09.006 19962494

[11] Kaplan CM, Kelleher E, Irani A, Schrepf A, Clauw DJ, Harte SE. Deciphering nociplastic pain: clinical features, risk factors and potential mechanisms. Nat Rev Neurol. 2024;20(6):347–63. doi: 10.1038/s41582-024-00966-8 38755449

[12] Mueller BR, Clauw DJ, De Lott LB. Advances in our understanding and treatment of nociplastic pain. Neurol Clin. 2025;43(3):549–60. doi: 10.1016/j.ncl.2025.04.005 40675665

[13] Gaskin DJ, Richard P. The economic costs of pain in the United States. J Pain. 2012;13(8):715–24. doi: 10.1016/j.jpain.2012.03.009 22607834

[14] Smith TJ, Hillner BE. The cost of pain. JAMA Network Open. 2019;2(4):e191532-e. doi: 10.1001/jamanetworkopen.2019.1532 30951152

[15] Hadi MA, McHugh GA, Closs SJ. Impact of chronic pain on patients’ quality of life: a comparative mixed-methods study. J Patient Exp. 2019;6(2):133–41. doi: 10.1177/2374373518786013 31218259 PMC6558939

[16] Hooten WM. Chronic pain and mental health disorders: shared neural mechanisms, epidemiology, and treatment. Mayo Clinic Proceedings. 2016;91(7):955–70. 27344405 10.1016/j.mayocp.2016.04.029

[17] Burke ALJ, Mathias JL, Denson LA. Psychological functioning of people living with chronic pain: a meta-analytic review. Br J Clin Psychol. 2015;54(3):345–60. doi: 10.1111/bjc.12078 25772553

[18] Reif S, Karriker-Jaffe KJ, Valentine A, Patterson D, Mericle AA, Adams RS, et al. Substance use and misuse patterns and disability status in the 2020 US National Alcohol Survey: a contributing role for chronic pain. Disabil Health J. 2022;15(2S):101290. doi: 10.1016/j.dhjo.2022.101290 35341718 PMC9232957

[19] Martel MO, Shir Y, Ware MA. Substance-related disorders: a review of prevalence and correlates among patients with chronic pain. Prog Neuropsychopharmacol Biol Psychiatry. 2018;87(Pt B):245–54. doi: 10.1016/j.pnpbp.2017.06.032 28669582

[20] Voon P, Greer AM, Amlani A, Newman C, Burmeister C, Buxton JA. Pain as a risk factor for substance use: a qualitative study of people who use drugs in British Columbia, Canada. Harm Reduct J. 2018;15(1):35. doi: 10.1186/s12954-018-0241-y 29976203 PMC6034304

[21] Edlund MJ, Sullivan MD, Han X, Booth BM. Days with pain and substance use disorders: is there an association? the clinical Journal of Pain. 2013;29(8). 10.1097/AJP.0b013e318270fa77 PMC3706984 23835765

[22] Wyse JJ, Lovejoy J, Holloway J, Morasco BJ, Dobscha SK, Hagedorn H, et al. Patients’ perceptions of the pathways linking chronic pain with problematic substance use. Pain. 2021;162(3):787–93. doi: 10.1097/j.pain.0000000000002077 32947546 PMC7886942

[23] Morasco BJ, Gritzner S, Lewis L, Oldham R, Turk DC, Dobscha SK. Systematic review of prevalence, correlates, and treatment outcomes for chronic non-cancer pain in patients with comorbid substance use disorder. Pain. 2011;152(3):488–97. doi: 10.1016/j.pain.2010.10.009 21185119 PMC3053013

[24] Trafton JA, Oliva EM, Horst DA, Minkel JD, Humphreys K. Treatment needs associated with pain in substance use disorder patients: implications for concurrent treatment. Drug Alcohol Depend. 2004;73(1):23–31. doi: 10.1016/j.drugalcdep.2003.08.007 14687956

[25] Turner HN, Oliver J, Compton P, Matteliano D, Sowicz TJ, Strobbe S, et al. Pain management and risks associated with substance use: practice recommendations. Pain Manag Nurs. 2022;23(2):91–108. doi: 10.1016/j.pmn.2021.11.002 34965906

[26] Savage SR, Kirsh KL, Passik SD. Challenges in using opioids to treat pain in persons with substance use disorders. Addict Sci Clin Pract. 2008;4(2):4–25. doi: 10.1151/ascp08424 18497713 PMC2797112

[27] Marshall B, Bland MK, Hulla R, Gatchel RJ. Considerations in addressing the opioid epidemic and chronic pain within the USA. Pain Manag. 2019;9(2):131–8. doi: 10.2217/pmt-2018-0070 30806566

[28] Volkow ND, McLellan AT. Opioid abuse in chronic pain — misconceptions and mitigation strategies. New England Journal of Medicine. 2016;374(13):1253–63. 27028915 10.1056/NEJMra1507771

[29] Vowles KE, McEntee ML, Julnes PS, Frohe T, Ney JP, van der Goes DN. Rates of opioid misuse, abuse, and addiction in chronic pain: a systematic review and data synthesis. Pain. 2015;156(4):569–76. doi: 10.1097/01.j.pain.0000460357.01998.f1 25785523

[30] Martinez-Calderon J, Flores-Cortes M, Morales-Asencio JM, Luque-Suarez A. Pain catastrophizing, opioid misuse, opioid use, and opioid dose in people with chronic musculoskeletal pain: a systematic review. The Journal of Pain. 2021;22(8):879–91. 33581324 10.1016/j.jpain.2021.02.002

[31] Papaleontiou M, Henderson CR, Turner BJ, Moore AA, Olkhovskaya Y, Amanfo L, et al. Outcomes associated with opioid use in the treatment of chronic noncancer pain in older adults: a systematic review and meta-analysis. J Am Geriatr Soc. 2010;58(7):1353–69. doi: 10.1111/j.1532-5415.2010.02920.x 20533971 PMC3114446

[32] Cragg A, Hau JP, Woo SA, Kitchen SA, Liu C, Doyle-Waters MM, et al. Risk factors for misuse of prescribed opioids: a systematic review and meta-analysis. Ann Emerg Med. 2019;74(5):634–46. doi: 10.1016/j.annemergmed.2019.04.019 31229388

[33] Delorme J, Kerckhove N, Authier N, Pereira B, Bertin C, Chenaf C. Systematic review and meta-analysis of the prevalence of chronic pain among patients with opioid use disorder and receiving opioid substitution therapy. J Pain. 2023;24(2):192–203. doi: 10.1016/j.jpain.2022.08.008 36220483

[34] Voon P, Karamouzian M, Kerr T. Chronic pain and opioid misuse: a review of reviews. Substance Abuse Treatment, Prevention, and Policy. 2017;12(1):36. 28810899 10.1186/s13011-017-0120-7 PMC5558770

[35] Lusted A, Roerecke M, Goldner E, Rehm J, Fischer B. Prevalence of pain among nonmedical prescription opioid users in substance use treatment populations: systematic review and meta-analyses. Pain Physician. 2013;16(6):E671-84. doi: 10.36076/ppj.2013/16/e671 24284850

[36] Dennis BB, Bawor M, Naji L, Chan CK, Varenbut J, Paul J, et al. Impact of chronic pain on treatment prognosis for patients with opioid use disorder: a systematic review and meta-analysis. Subst Abuse. 2015;9:59–80. doi: 10.4137/SART.S30120 26417202 PMC4573077

[37] Witkiewitz K, Vowles KE. Alcohol and opioid use, co-use, and chronic pain in the context of the opioid epidemic: a critical review. Alcoholism: Clinical and Experimental Research. 2018;42(3):478–88. 29314075 10.1111/acer.13594 PMC5832605

[38] Ditre JW, Brandon TH, Zale EL, Meagher MM. Pain, nicotine, and smoking: research findings and mechanistic considerations. Psychol Bull. 2011;137(6):1065–93. doi: 10.1037/a0025544 21967450 PMC3202023

[39] Ditre JW, Zale EL, LaRowe LR. A reciprocal model of pain and substance use: transdiagnostic considerations, clinical implications, and future directions. Annu Rev Clin Psychol. 2019;15:503–28. doi: 10.1146/annurev-clinpsy-050718-095440 30566371

[40] Zale EL, Maisto SA, Ditre JW. Interrelations between pain and alcohol: an integrative review. Clin Psychol Rev. 2015;37:57–71. doi: 10.1016/j.cpr.2015.02.005 25766100 PMC4385458

[41] Ferguson E, Zale E, Ditre J, Wesolowicz D, Stennett B, Robinson M, et al. CANUE: A Theoretical Model of Pain as an Antecedent for Substance Use. Ann Behav Med. 2021;55(5):489–502. doi: 10.1093/abm/kaaa072 32914834 PMC8122475

[42] Baker TB, Piper ME, McCarthy DE, Majeskie MR, Fiore MC. Addiction motivation reformulated: an affective processing model of negative reinforcement. Psychological Review. 2004;111(1):33–51. 14756584 10.1037/0033-295X.111.1.33

[43] Zvolensky MJ, Cougle JR, Bonn-Miller MO, Norberg MM, Johnson K, Kosiba J, et al. Chronic pain and marijuana use among a nationally representative sample of adults. Am J Addict. 2011;20(6):538–42. doi: 10.1111/j.1521-0391.2011.00176.x 21999500

[44] Zvolensky MJ, McMillan KA, Gonzalez A, Asmundson GJG. Chronic musculoskeletal pain and cigarette smoking among a representative sample of Canadian adolescents and adults. Addict Behav. 2010;35(11):1008–12. doi: 10.1016/j.addbeh.2010.06.019 20621422 PMC2919644

[45] McDermott KA, Joyner KJ, Hakes JK, Okey SA, Cougle JR. Pain interference and alcohol, nicotine, and cannabis use disorder in a national sample of substance users. Drug Alcohol Depend. 2018;186:53–9. doi: 10.1016/j.drugalcdep.2018.01.011 29550622

[46] Brennan PL, Schutte KK, SooHoo S, Moos RH. Painful medical conditions and alcohol use: a prospective study among older adults. Pain Med. 2011;12(7):1049–59. doi: 10.1111/j.1526-4637.2011.01156.x 21668742 PMC3146463

[47] Brennan PL, Soohoo S. Pain and use of alcohol in later life: prospective evidence from the health and retirement study. J Aging Health. 2013;25(4):656–77. doi: 10.1177/0898264313484058 23640817 PMC3883439

[48] Rehm J, Marmet S, Anderson P, Gual A, Kraus L, Nutt D. Defining substance use disorders: do we really need more than heavy use?. Alcohol and Alcoholism. 2013;48(6):633–40. 23926213 10.1093/alcalc/agt127

[49] Kosek E, Clauw D, Nijs J, Baron R, Gilron I, Harris RE, et al. Chronic nociplastic pain affecting the musculoskeletal system: clinical criteria and grading system. Pain. 2021;162(11):2629–34. doi: 10.1097/j.pain.0000000000002324 33974577

[50] Maixner W, Fillingim RB, Williams DA, Smith SB, Slade GD. Overlapping chronic pain conditions: implications for diagnosis and classification. J Pain. 2016;17(9 Suppl):T93–107. doi: 10.1016/j.jpain.2016.06.002 27586833 PMC6193199

[51] Larance B, Campbell G, Peacock A, Nielsen S, Bruno R, Hall W, et al. Pain, alcohol use disorders and risky patterns of drinking among people with chronic non-cancer pain receiving long-term opioid therapy. Drug Alcohol Depend. 2016;162:79–87. doi: 10.1016/j.drugalcdep.2016.02.048 27049582

[52] Boissoneault J, Lewis B, Nixon SJ. Characterizing chronic pain and alcohol use trajectory among treatment-seeking alcoholics. Alcohol. 2019;75:47–54. doi: 10.1016/j.alcohol.2018.05.009 30359794

[53] Boehnke KF, Scott JR, Litinas E, Sisley S, Williams DA, Clauw DJ. High-frequency medical cannabis use is associated with worse pain among individuals with chronic pain. J Pain. 2020;21(5–6):570–81. 31560957 10.1016/j.jpain.2019.09.006 PMC7089844

[54] Goesling J, Brummett CM, Meraj TS, Moser SE, Hassett AL, Ditre JW. Associations between pain, current tobacco smoking, depression, and fibromyalgia status among treatment-seeking chronic pain patients. Pain Med. 2015;16(7):1433–42. doi: 10.1111/pme.12747 25801019 PMC4765172

[55] Mannes ZL, Malte CA, Olfson M, Wall MM, Keyes KM, Martins SS, et al. Increasing risk of cannabis use disorder among U.S. veterans with chronic pain: 2005-2019. Pain. 2023;164(9):2093–103. doi: 10.1097/j.pain.0000000000002920 37159542 PMC10524371

[56] Bialas P, Böttge-Wolpers C, Fitzcharles M-A, Gottschling S, Konietzke D, Juckenhöfel S, et al. Cannabis use disorder in patients with chronic pain: overestimation and underestimation in a cross-sectional observational study in 3 German pain management centres. Pain. 2023;164(6):1303–11. doi: 10.1097/j.pain.0000000000002817 36327134

[57] Shamseer L, Moher D, Clarke M, Ghersi D, Liberati A, Petticrew M, et al. Preferred reporting items for systematic review and meta-analysis protocols (PRISMA-P) 2015: elaboration and explanation. BMJ. 2015;350:g7647. doi: 10.1136/bmj.g7647 25555855

[58] Munn Z, Moola S, Lisy K, Riitano D, Tufanaru C. Methodological guidance for systematic reviews of observational epidemiological studies reporting prevalence and cumulative incidence data. Int J Evid Based Healthc. 2015;13(3):147–53. doi: 10.1097/XEB.0000000000000054 26317388

[59] Peters JL, Sutton AJ, Jones DR, Abrams KR, Rushton L. Contour-enhanced meta-analysis funnel plots help distinguish publication bias from other causes of asymmetry. J Clin Epidemiol. 2008;61(10):991–6. doi: 10.1016/j.jclinepi.2007.11.010 18538991

[60] Egger M, Davey Smith G, Schneider M, Minder C. Bias in meta-analysis detected by a simple, graphical test. BMJ. 1997;315(7109):629–34. doi: 10.1136/bmj.315.7109.629 9310563 PMC2127453

[61] Duval S, Tweedie R. Trim and fill: A simple funnel-plot-based method of testing and adjusting for publication bias in meta-analysis. Biometrics. 2000;56(2):455–63. doi: 10.1111/j.0006-341x.2000.00455.x 10877304

[62] Zale EL, Lange KL, Fields SA, Ditre JW. The relation between pain-related fear and disability: a meta-analysis. J Pain. 2013;14(10):1019–30. doi: 10.1016/j.jpain.2013.05.005 23850095 PMC3791167

[63] Barendregt JJ, Doi SA, Lee YY, Norman RE, Vos T. Meta-analysis of prevalence. J Epidemiol Community Health. 2013;67(11):974–8. doi: 10.1136/jech-2013-203104 23963506

[64] Rotenstein LS, Ramos MA, Torre M, Segal JB, Peluso MJ, Guille C, et al. Prevalence of depression, depressive symptoms, and suicidal ideation among medical students: a systematic review and meta-analysis. JAMA. 2016;316(21):2214–36. doi: 10.1001/jama.2016.17324 27923088 PMC5613659

[65] Borenstein M, Higgins JPT. Meta-analysis and subgroups. Prev Sci. 2013;14(2):134–43. doi: 10.1007/s11121-013-0377-7 23479191

[66] Sterne JAC, Jüni P, Schulz KF, Altman DG, Bartlett C, Egger M. Statistical methods for assessing the influence of study characteristics on treatment effects in “meta-epidemiological” research. Stat Med. 2002;21(11):1513–24. doi: 10.1002/sim.1184 12111917

[67] van Houwelingen HC, Arends LR, Stijnen T. Advanced methods in meta-analysis: multivariate approach and meta-regression. Stat Med. 2002;21(4):589–624. doi: 10.1002/sim.1040 11836738

[68] Wood JA. Methodology for dealing with duplicate study effects in a meta-analysis. Organizational Research Methods. 2007;11(1):79–95. doi: 10.1177/1094428106296638

[69] Partlett C, Riley RD. Random effects meta-analysis: coverage performance of 95% confidence and prediction intervals following REML estimation. Stat Med. 2017;36(2):301–17. doi: 10.1002/sim.7140 27714841 PMC5157768

[70] Siegel LK, Silva M, Lin L, Chen Y, Liu Y-L, Chu H. Choice of link functions for generalized linear mixed models in meta-analyses of proportions. Res Methods Med Health Sci. 2025;6(1):13–23. doi: 10.1177/26320843231224808 39668973 PMC11632795

[71] Lin L, Chu H. Meta-analysis of proportions using generalized linear mixed models. Epidemiology. 2020;31(5):713–7. doi: 10.1097/EDE.0000000000001232 32657954 PMC7398826

[72] Migliavaca CB, Stein C, Colpani V, Barker TH, Ziegelmann PK, Munn Z, et al. Meta-analysis of prevalence: I2 statistic and how to deal with heterogeneity. Res Synth Methods. 2022;13(3):363–7. doi: 10.1002/jrsm.1547 35088937

[73] Lin L. Comparison of four heterogeneity measures for meta-analysis. J Eval Clin Pract. 2020;26(1):376–84. doi: 10.1111/jep.13159 31234230

[74] Melsen W, Bootsma M, Rovers M, Bonten M. The effects of clinical and statistical heterogeneity on the predictive values of results from meta-analyses. Clinical Microbiology and Infection. 2014;20(2):123–9. 24320992 10.1111/1469-0691.12494

[75] Campbell M, McKenzie JE, Sowden A, Katikireddi SV, Brennan SE, Ellis S, et al. Synthesis without meta-analysis (SWiM) in systematic reviews: reporting guideline. BMJ. 2020;368:l6890. doi: 10.1136/bmj.l6890 31948937 PMC7190266

[76] Higgins JPT, Thompson SG. Controlling the risk of spurious findings from meta-regression. Stat Med. 2004;23(11):1663–82. doi: 10.1002/sim.1752 15160401

[77] Valentine JC, Pigott TD, Rothstein HR. How many studies do you need? A primer on statistical power for meta-analysis. Journal of Educational and Behavioral Statistics. 2010;35(2):215–47.

[78] Jackson D, Turner R. Power analysis for random-effects meta-analysis. Res Synth Methods. 2017;8(3):290–302. doi: 10.1002/jrsm.1240 28378395 PMC5590730

[79] Grigsby TJ. Leaving “Drug abuse” behind: a theoretical and methodological heuristic to selecting “problem drug use” or “drug misuse” as alternative terms. Subst Use Misuse. 2021;56(13):2074–7. doi: 10.1080/10826084.2021.1963989 34404316

[80] Mahmoud KF, Finnell D, Savage CL, Puskar KR, Mitchell AM. A concept analysis of substance misuse to inform contemporary terminology. Arch Psychiatr Nurs. 2017;31(6):532–40. doi: 10.1016/j.apnu.2017.06.004 29179817

[81] Volkow ND, Gordon JA, Koob GF. Choosing appropriate language to reduce the stigma around mental illness and substance use disorders. Neuropsychopharmacology. 2021;46(13):2230–2. doi: 10.1038/s41386-021-01069-4 34276051 PMC8580983

[82] Ashford RD, Brown AM, McDaniel J, Curtis B. Biased labels: an experimental study of language and stigma among individuals in recovery and health professionals. Subst Use Misuse. 2019;54(8):1376–84. doi: 10.1080/10826084.2019.1581221 30945955 PMC6510618

[83] Marino EN, Jha MK, Minhajuddin A, Ayvaci ER, Levinson S, Pipes R, et al. Problematic substance use in depressed adolescents: prevalence and clinical correlates. Addict Behav Rep. 2024;19:100539. doi: 10.1016/j.abrep.2024.100539 38510109 PMC10951442

[84] Shenoi RP, Linakis JG, Bromberg JR, Casper TC, Richards R, Mello MJ, et al. Predictive validity of the CRAFFT for substance use disorder. Pediatrics. 2019;144(2):e20183415. doi: 10.1542/peds.2018-3415 31341007 PMC6855834

[85] Steele DW, Becker SJ, Danko KJ, Balk EM, Adam GP, Saldanha IJ, et al. Brief behavioral interventions for substance use in adolescents: a meta-analysis. Pediatrics. 2020;146(4):e20200351. doi: 10.1542/peds.2020-0351 32928988

