# Supplementary material for: Prevalence of nicotine, alcohol, and cannabis use disorders among adults with chronic pain: Protocol for a systematic review and meta-analysis
Source: PLoS One. 2026 Aug 26;21(8):e0356365. doi: 10.1371/journal.pone.0356365 (PMC13514021; doi:10.1371/journal.pone.0356365)
Supplement: Supplementary file 1 [file pone.0356365.s001.docx]

| Table 1. Inclusion and Exclusion Criteria for Reviewed Studies | |
| --- | --- |
| **Inclusion criteria** | **Exclusion criteria** |
| - Adults at least 18 years of age - Chronic non-cancer pain   - Diagnosed chronic pain condition by a clinician, medical record review, validated self-report, or self-reported pain for ≥ 3 months - Reporting past-year and/or lifetime substance use disorder or dependence (i.e. alcohol, nicotine, and cannabis)   - Diagnoses must be based upon any version of the ICD or DSM criteria, assessed via clinical examination or interviews by using validated instruments.   - Studies using screening tools will be included if the instrument has been validated against any version of the DSM or ICD criteria   OR   - Reporting problematic (abuse/misuse/hazardous) alcohol, nicotine, and/or cannabis use over the past 12 months   - As determined by validated screening instruments with pre-defined cutoffs (e.g., Alcohol Use Disorders Identified Test, Cannabis Use Disorders Identification Test). | - Cancer-related pain as the only pain reported - Studies on nicotine replacement therapy - Case reports, review articles, opinion pieces, commentaries, editorials, conference abstracts, and protocols - Studies focused exclusively on opioid use (non-opioid substance use data are not reported) |
