# Supplementary material for: Prevalence of nicotine, alcohol, and cannabis use disorders among adults with chronic pain: Protocol for a systematic review and meta-analysis
Source: PLoS One. 2026 Aug 26;21(8):e0356365. doi: 10.1371/journal.pone.0356365 (PMC13514021; doi:10.1371/journal.pone.0356365)
Supplement: Supplementary file 2 [file pone.0356365.s002.docx]

**Table 2. Terminology referring to problematic substance use**

| **Term** | **Brief definition / note** |
| --- | --- |
| **Problematic substance use** | Broad, nonjudgmental term for substance use associated multiple outcomes, including harm, impairment, elevated risk, or other clinically relevant adverse consequences. Indicative of clinically meaningful use-related problems that do not meet disorder criteria. |
| **Unhealthy substance use** | Refers to substance use that increases the likelihood of adverse health outcomes, whether or not severe consequences are already present. |
| **Risky substance use** | Describes a pattern of use associated with increased probability of harm. |
| **Hazardous substance use** | Refers to use that places an individual at increased risk of harmful consequences, that is typically operationally defined by screening measure (e.g., hazardous alcohol use). |
| **Harmful substance use** | Describes substance use already associated with physical, psychological, or social harm. |
| **Substance misuse** | Commonly used to describe substance use in a harmful, excessive, or unintended manner. Often used in the literature and also may be imprecise in describing use and associated problems. |
| **Nonmedical use** | Refers to use of a medication outside its intended medical purpose, such as without a prescription, in greater amounts, or for reasons other than prescribed. |
| **Substance-related harm** | Emphasizes adverse consequences attributable to substance use, including health, social, occupational, or functional harms. |
