# Supplementary material for: Prevalence of nicotine, alcohol, and cannabis use disorders among adults with chronic pain: Protocol for a systematic review and meta-analysis
Source: PLoS One. 2026 Aug 26;21(8):e0356365. doi: 10.1371/journal.pone.0356365 (PMC13514021; doi:10.1371/journal.pone.0356365)
Supplement: Supplementary file 3 [file pone.0356365.s004.docx]

**APPENDIX 1: PubMed Search Strategy**

# Chronic pain SR PubMed search

(("vulvodynia"[Title/Abstract] OR "vulva pain"[Title/Abstract] OR "vulvodynia"[MeSH Terms] OR "endometriosis"[Title/Abstract] OR "endometriosis"[MeSH Terms] OR ("temporomandibular"[Title/Abstract] AND ("disease"[Title/Abstract] OR "diseases"[Title/Abstract] OR "disorder"[Title/Abstract] OR "disorders"[Title/Abstract])) OR "tmj"[Title/Abstract] OR "temporomandibular joint disorders"[MeSH Terms] OR "myalgic encephalomyelitis"[Title/Abstract] OR "chronic fatigue"[Title/Abstract] OR "fatigue syndrome, chronic"[MeSH Terms] OR "irritable bowel syndrome"[Title/Abstract] OR "irritable bowel syndromes"[Title/Abstract] OR "irritable bowel syndrome"[MeSH Terms] OR "interstitial cystitis"[Title/Abstract] OR "painful bladder syndrome"[Title/Abstract] OR "cystitis, interstitial"[MeSH Terms] OR "fibromyalgia"[Title/Abstract] OR "fibromyalgias"[Title/Abstract] OR "fibromyalgia"[MeSH Terms] OR "low back pain"[Title/Abstract] OR "lower back pain"[Title/Abstract] OR "low back pain"[MeSH Terms] OR "headache"[Title/Abstract] OR "headaches"[Title/Abstract] OR "headache disorders"[MeSH Terms] OR "migraine"[Title/Abstract] OR "migraines"[Title/Abstract] OR ("chronic"[Title/Abstract] AND "pain"[Title/Abstract]) OR "chronic pain"[MeSH Terms] OR "recurrent pain"[Title/Abstract] OR "intractable pain"[Title/Abstract] OR "refractory pain"[Title/Abstract] OR "persistent pain"[Title/Abstract] OR "pain, intractable"[MeSH Terms] OR "musculoskeletal pain"[Title/Abstract] OR "musculoskeletal pain"[MeSH Terms] OR "neuropathic pain"[Title/Abstract] OR "neuralgia"[Title/Abstract] OR "neuralgia"[MeSH Terms] OR "arthritis"[Title/Abstract] OR "osteoarthritis"[Title/Abstract] OR "arthritis"[MeSH Terms] OR "pelvic pain"[Title/Abstract] OR "pelvic pain"[MeSH Terms] OR "neuralgias"[Title/Abstract] OR "painful bladder syndromes"[Title/Abstract]) AND ((("alcohol"[Title/Abstract] OR "beer"[Title/Abstract] OR "wine"[Title/Abstract] OR "drink"[Title/Abstract] OR "drinks"[Title/Abstract] OR "drinker"[Title/Abstract] OR "drinkers"[Title/Abstract] OR "drinking"[Title/Abstract] OR "drank"[Title/Abstract] OR "drunk"[Title/Abstract] OR "alcohol drinking"[MeSH Terms] OR "smoking"[Title/Abstract] OR "smoker"[Title/Abstract] OR "smokers"[Title/Abstract] OR "smoke"[Title/Abstract] OR "smokes"[Title/Abstract] OR "smoked"[Title/Abstract] OR "vape"[Title/Abstract] OR "vaped"[Title/Abstract] OR "vaping"[Title/Abstract] OR "hookah"[Title/Abstract] OR "hookahs"[Title/Abstract] OR ("smoking"[MeSH Terms] NOT "cocaine smoking"[MeSH Terms]) OR "cigar"[Title/Abstract] OR "cigars"[Title/Abstract] OR "cigarette"[Title/Abstract] OR "cigarettes"[Title/Abstract] OR "tobacco"[Title/Abstract] OR "smokers"[MeSH Terms] OR "tobacco use"[MeSH Terms] OR "nicotine"[Title/Abstract] OR "nicotine"[MeSH Terms] OR "smoking devices"[MeSH Terms] OR "cannabis"[Title/Abstract] OR "marijuana"[Title/Abstract] OR "hashish"[Title/Abstract] OR "hash"[Title/Abstract] OR "weed"[Title/Abstract] OR "cannabis"[MeSH Terms] OR "marijuana use"[MeSH Terms] OR "cannabinoid"[Title/Abstract] OR "cannabinoids"[Title/Abstract] OR "cannabinol"[Title/Abstract] OR "dronabinol"[Title/Abstract] OR "cannabinoids"[MeSH Terms] OR "vapes"[Title/Abstract]) AND ("abuse"[Title/Abstract] OR "abuses"[Title/Abstract] OR "abused"[Title/Abstract] OR "abusing"[Title/Abstract] OR "abuser"[Title/Abstract] OR "abusers"[Title/Abstract] OR "addict"[Title/Abstract] OR "addicts"[Title/Abstract] OR "addicted"[Title/Abstract] OR "addiction"[Title/Abstract] OR "addictions"[Title/Abstract] OR "binge"[Title/Abstract] OR "daily"[Title/Abstract] OR "dependent"[Title/Abstract] OR "dependence"[Title/Abstract] OR "excessive"[Title/Abstract] OR "excessively"[Title/Abstract] OR "harmful"[Title/Abstract] OR "heavy"[Title/Abstract] OR "heavily"[Title/Abstract] OR "misuse"[Title/Abstract] OR "misused"[Title/Abstract] OR "misuses"[Title/Abstract] OR "misusing"[Title/Abstract] OR "overconsumption"[Title/Abstract] OR "overconsume"[Title/Abstract] OR "overconsumes"[Title/Abstract] OR "overconsumed"[Title/Abstract] OR "overconsuming"[Title/Abstract] OR "problem"[Title/Abstract] OR "problematic"[Title/Abstract] OR "risky"[Title/Abstract] OR "use disorder"[Title/Abstract] OR "use disorders"[Title/Abstract])) OR ("alcoholic"[Title/Abstract] OR "alcoholics"[Title/Abstract] OR "alcoholism"[Title/Abstract] OR "alcoholics"[MeSH Terms] OR "Alcohol-Related Disorders"[Mesh:NoExp] OR "alcoholic intoxication"[MeSH Terms] OR "alcoholism"[MeSH Terms] OR "binge drinking"[MeSH Terms] OR "tobacco use disorder"[MeSH Terms] OR "Marijuana Abuse"[Mesh:NoExp])))
