# Supplementary material for: Prevalence of nicotine, alcohol, and cannabis use disorders among adults with chronic pain: Protocol for a systematic review and meta-analysis
Source: PLoS One. 2026 Aug 26;21(8):e0356365. doi: 10.1371/journal.pone.0356365 (PMC13514021; doi:10.1371/journal.pone.0356365)
Supplement: Peer review history

# Peer review: Decision Letter 0

**DOI:** 10.1371/journal.pone.0356365.r001

**Authors:** Marianna Mazza

17 Feb 2026

Dear Dr.  Rogers,

Thank you for submitting your manuscript to PLOS ONE. After careful consideration, we feel that it has merit but does not fully meet PLOS ONE’s publication criteria as it currently stands. Therefore, we invite you to submit a revised version of the manuscript that carefully addresses the points raised by the three reviewers.

Please submit your revised manuscript by Apr 03 2026 11:59PM. If you will need more time than this to complete your revisions, please reply to this message or contact the journal office at plosone@plos.org. When you're ready to submit your revision, log on to https://www.editorialmanager.com/pone/ and select the 'Submissions Needing Revision' folder to locate your manuscript file.

- A letter that responds to each point raised by the academic editor and reviewer(s). You should upload this letter as a separate file labeled 'Response to Reviewers'.
- A marked-up copy of your manuscript that highlights changes made to the original version. You should upload this as a separate file labeled 'Revised Manuscript with Track Changes'.
- An unmarked version of your revised paper without tracked changes. You should upload this as a separate file labeled 'Manuscript'.

If you would like to make changes to your financial disclosure, please include your updated statement in your cover letter. Guidelines for resubmitting your figure files are available below the reviewer comments at the end of this letter.

If applicable, we recommend that you deposit your laboratory protocols in protocols.io to enhance the reproducibility of your results. Protocols.io assigns your protocol its own identifier (DOI) so that it can be cited independently in the future. For instructions see: https://journals.plos.org/plosone/s/submission-guidelines#loc-laboratory-protocols. Additionally, PLOS ONE offers an option for publishing peer-reviewed Lab Protocol articles, which describe protocols hosted on protocols.io. Read more information on sharing protocols at https://plos.org/protocols?utm_medium=editorial-email&utm_source=authorletters&utm_campaign=protocols.

We look forward to receiving your revised manuscript.

Kind regards,

Marianna Mazza

Academic Editor

PLOS One

Journal Requirements:

When submitting your revision, we need you to address these additional requirements.

1.Please ensure that your manuscript meets PLOS ONE's style requirements, including those for file naming. The PLOS ONE style templates can be found at

https://journals.plos.org/plosone/s/file?id=wjVg/PLOSOne_formatting_sample_main_body.pdf and

https://journals.plos.org/plosone/s/file?id=ba62/PLOSOne_formatting_sample_title_authors_affiliations.pdf

2. Please provide a complete Data Availability Statement in the submission form, ensuring you include all necessary access information or a reason for why you are unable to make your data freely accessible. If your research concerns only data provided within your submission, please write "All data are in the manuscript and/or supporting information files" as your Data Availability Statement.

3. When completing the data availability statement of the submission form, you indicated that you will make your data available on acceptance. We strongly recommend all authors decide on a data sharing plan before acceptance, as the process can be lengthy and hold up publication timelines. Please note that, though access restrictions are acceptable now, your entire data will need to be made freely accessible if your manuscript is accepted for publication. This policy applies to all data except where public deposition would breach compliance with the protocol approved by your research ethics board. If you are unable to adhere to our open data policy, please kindly revise your statement to explain your reasoning and we will seek the editor's input on an exemption. Please be assured that, once you have provided your new statement, the assessment of your exemption will not hold up the peer review process.

4. Please include captions for your Supporting Information files at the end of your manuscript, and update any in-text citations to match accordingly. Please see our Supporting Information guidelines for more information: http://journals.plos.org/plosone/s/supporting-information.

5. If the reviewer comments include a recommendation to cite specific previously published works, please review and evaluate these publications to determine whether they are relevant and should be cited. There is no requirement to cite these works unless the editor has indicated otherwise.

[Note: HTML markup is below. Please do not edit.]

Reviewer's Responses to Questions

**Comments to the Author**

1. Does the manuscript provide a valid rationale for the proposed study, with clearly identified and justified research questions?

Reviewer #1: Yes

Reviewer #2: Yes

Reviewer #3: Partly

**********

2. Is the protocol technically sound and planned in a manner that will lead to a meaningful outcome and allow testing the stated hypotheses?

Reviewer #1: Partly

Reviewer #2: Yes

Reviewer #3: Partly

**********

3. Is the methodology feasible and described in sufficient detail to allow the work to be replicable?

Reviewer #1: Yes

Reviewer #2: Yes

Reviewer #3: No

**********

4. Have the authors described where all data underlying the findings will be made available when the study is complete?

The PLOS Data policy

Reviewer #1: No

Reviewer #2: Yes

Reviewer #3: Yes

**********

5. Is the manuscript presented in an intelligible fashion and written in standard English?

Reviewer #1: Yes

Reviewer #2: Yes

Reviewer #3: Yes

**********

Please use the space provided to explain your answers to the questions above and, if applicable, provide comments about issues authors must address before this protocol can be accepted for publication. You may also include additional comments for the author, including concerns about research or publication ethics.

You may also provide optional suggestions and comments to authors that they might find helpful in planning their study.

Reviewer #1: Comments to the Authors

Major Comments

1. Conceptual Clarity of “Problematic Substance Use”

The protocol appropriately acknowledges heterogeneity in how problematic substance use is defined; however, the current operationalization remains overly broad.

The inclusion criteria combine:

Hazardous/problematic use (screening-based),

Diagnosed substance use disorders (DSM/ICD),

Past-year and lifetime measures.

These constructs reflect distinct clinical severities and time frames, yet are pooled under a single analytic framework.

Suggestions:

Explicitly predefine tiers or categories of problematic use (e.g., hazardous use vs. diagnosed disorder).

Clarify whether pooled estimates will be stratified by:

Diagnostic vs. screening-based definitions,

Past-year vs. lifetime prevalence.

Consider whether combining these constructs risks inflating or obscuring clinically meaningful prevalence estimates.

2. Decision to Average Multiple Prevalence Estimates Within Studies

The protocol states that when studies report multiple prevalence estimates for the same substance, a mean prevalence rate will be calculated.

This approach is methodologically concerning, as it:

Violates assumptions of independence,

Masks subgroup variability,

May introduce bias depending on subgroup sizes.

Suggestions:

Justify this approach more explicitly with citations from prevalence meta-analysis methodology.

Consider alternative strategies, such as:

Selecting the most clinically relevant estimate (e.g., total sample),

Conducting sensitivity analyses,

Using multilevel or robust variance meta-analytic approaches.

3. Anticipated Heterogeneity and Its Implications

Given the wide range of:

Chronic pain definitions,

Pain conditions,

Substance types, products, and routes of administration,

Measurement tools,

very high heterogeneity is likely.

Suggestions:

Elaborate on how extreme heterogeneity (e.g., I² > 90%) will be handled in interpretation.

Clarify whether pooled estimates will still be presented under such conditions, or whether narrative synthesis thresholds will be applied.

Consider pre-specifying criteria for when meta-analysis may be deemed inappropriate.

4. Exclusion of Non-English Full-Text Articles

While resource constraints are understandable, excluding non-English full texts may bias prevalence estimates, particularly given global variation in chronic pain and substance use.

Suggestions:

Explicitly acknowledge this as a source of language and regional bias in both the Methods and Discussion.

Clarify whether non-English abstracts will contribute to any descriptive mapping of excluded studies (beyond listing in an appendix).

5. Co-use of Substances

The protocol states that studies reporting co-use will be included, but the analytic handling of co-use is unclear.

Suggestions:

Clarify whether co-use prevalence will be:

Reported descriptively,

Analyzed separately,

Or excluded from pooled estimates.

Explicitly state whether individuals may be counted multiple times across substance-specific analyses.

Minor Comments

Terminology consistency

The manuscript alternates between “problematic use,” “misuse,” “hazardous use,” and “use disorder.”

Recommend standardizing terminology and providing a brief definitional table.

Grammar and clarity

Minor typographical issues (e.g., duplicated phrases such as “full the full searches”) should be corrected.

Some long sentences in the Introduction could be tightened for clarity.

Data Availability Statement

While acceptable for a protocol, the statement could be strengthened by briefly outlining where and how extracted data and analytic code will be shared upon completion (e.g., OSF, GitHub).

Table 1

Consider explicitly stating whether mixed pain populations (e.g., cancer + non-cancer pain) are excluded or handled separately.

Overall Recommendation

This is a well-motivated and potentially impactful protocol, addressing an important and understudied area at the intersection of chronic pain and non-opioid substance use. With clearer conceptual definitions, stronger justification for analytic decisions, and improved handling of heterogeneity, the manuscript would meet high methodological standards and provide a solid foundation for the planned review.

Recommendation: Major revision

Reviewer #2: This protocol addresses a critical and timely gap in the literature regarding the prevalence of problematic nicotine, alcohol, and cannabis use among adults with chronic pain. While opioid use has been extensively characterized, the scope of non-opioid substance use in this population remains less precisely defined. The protocol is methodologically strong overall, adheres to PRISMA-P guidance, and outlines a thoughtful statistical plan. The study aims are well aligned with the stated need to better quantify prevalence estimates in this area.

That said, some technical aspects related to the search strategy and data synthesis would benefit from additional clarification to ensure the highest standards of transparency and reproducibility.

1. Search Strategy Refinement – Seed Article Omission

The protocol notes that one seed article was not retrieved because it lacked “misuse” terminology. This raises the possibility that the current search string may be overly restrictive. It would be helpful to clarify whether the search strategy was subsequently modified to incorporate broader terminology (e.g., high-risk use, heavy use, or consumption-level descriptors), or whether the decision reflects an intentional prioritization of specificity over sensitivity.

2. Database Translation and Language Bias

The protocol indicates that articles may be excluded if translation is not feasible due to funding limitations. This introduces the potential for language and geographic bias. It would strengthen the protocol to explicitly state how this limitation will be reported in the final manuscript and whether machine translation tools will be used for preliminary screening or data extraction feasibility assessment.

3. Operationalization of “Problematic Use”

The protocol plans to estimate prevalence for both “problematic use” and “use disorders.” Given the variability in how problematic use is defined across studies (for example, frequency-based thresholds versus consequence-based definitions), it would be valuable to provide a predefined hierarchical or decision framework for categorizing heterogeneous measures during data extraction. This would help ensure consistency across studies and improve interpretability of pooled estimates.

4. Statistical Rigor and Handling of Heterogeneity

The requirement of at least five studies to conduct random-effects meta-analysis is a commendably conservative decision. The approach of averaging prevalence rates derived from the same sample is also appropriate. For subgroup and moderator analyses, additional clarification would be helpful regarding how studies reporting multiple substances or co-use within the same population will be handled to avoid unit-of-analysis issues.

5. Risk of Bias Integration

The use of the Joanna Briggs Institute checklist for prevalence data is well justified. It would further strengthen the analytic plan to specify whether risk-of-bias scores will be incorporated as moderators or sensitivity analyses, rather than used solely for descriptive reporting.

6. Inclusion Criteria Clarification (Table 1)

The inclusion of self-reported pain ≥3 months is appropriate and consistent with standard chronic pain definitions. It may be helpful to explicitly state in the text (not only in the table) whether studies focused exclusively on cancer-related pain will be excluded, to reinforce the focus on chronic non-cancer pain.

7. Terminology in the Introduction

In the sentence stating that a systematic review and meta-analysis “conducted in 2011” reported prevalence estimates, it may be more precise to use “published in 2011,” since the cited year reflects publication rather than study conduct.

8. Minor Editorial Issue

In the Materials and Methods section, the phrase “full the full searches” appears to contain a duplicated word and should be corrected for clarity.

Overall, this is a well-conceived and clinically important protocol. Addressing the points above would further strengthen methodological transparency and reproducibility and improve clarity for readers and future replication efforts.

Reviewer #3: The manuscript needs the following clarifications:

1. The title ‘use disorders’ is creating confusion. How does it matter if this term is deleted?

2. It would be much better to show the entire research question into a PICO format.

3. Line 159: “cohort studies (both non-randomized and randomized controlled trials)” – RCT and NRCT are not part of a cohort study – please correct.

4. Objective is to find out the ‘estimate’ – does it include only prevalence? Since, the term prevalence has been used in the introduction section. Then, cohort studies might not be needed. If incidence is also there, then the rationality of cohort studies is justified. Moreover, RCT and NRCT will not provide any estimate of any of the use. So, please correct or clarify the entire manuscript.

5. Scopus, being the largest database, is not considered – why?

6. Line 174: For the same reason as mentioned earlier, clinical trials might not be suited.

7. Whether sensitivity analysis will be done?

8. It would be very helpful to the readers if the authors could highlight the high heterogeneity, how it would influence the interpretation of pooled prevalence estimates and mention what could be the possible reasons for the high heterogeneity.

9. Please mention potential confounding variables that may influence the findings.

10. Conclusion: The conclusion can be strengthened by adding 1 or 2 actionable steps for the stakeholders.

**********

what does this mean?). If published, this will include your full peer review and any attached files.

If you choose “no”, your identity will remain anonymous but your review may still be made public.

**Do you want your identity to be public for this peer review?**  For information about this choice, including consent withdrawal, please see our Privacy Policy

Reviewer #1: No

Reviewer #2: No

Reviewer #3: **Yes:** Indranil Saha

**********

[NOTE: If reviewer comments were submitted as an attachment file, they will be attached to this email and accessible via the submission site. Please log into your account, locate the manuscript record, and check for the action link "View Attachments". If this link does not appear, there are no attachment files.]

To ensure your figures meet our technical requirements, please review our figure guidelines: https://journals.plos.org/plosone/s/figures

You may also use PLOS’s free figure tool, NAAS, to help you prepare publication quality figures: https://journals.plos.org/plosone/s/figures#loc-tools-for-figure-preparation.

NAAS will assess whether your figures meet our technical requirements by comparing each figure against our figure specifications.

# Author comment: Author response to Decision Letter 1

**DOI:** 10.1371/journal.pone.0356365.r002

8 May 2026

Response Letter to Reviewers

We appreciate the thoughtful review and the opportunity to revise and resubmit this manuscript. We believe the suggested revisions have significantly strengthened the manuscript. In this letter, we outline how we addressed the feedback provided by reviewers. For ease of review, we have highlighted the corresponding changes in the manuscript in yellow.

Reviewer #1

Major Comments

1. Conceptual Clarity of “Problematic Substance Use”: The protocol appropriately acknowledges heterogeneity in how problematic substance use is defined; however, the current operationalization remains overly broad. The inclusion criteria combine: Hazardous/problematic use (screening-based), Diagnosed substance use disorders (DSM/ICD), Past-year and lifetime measures. These constructs reflect distinct clinical severities and time frames yet are pooled under a single analytic framework.

Suggestions:

• Explicitly predefine tiers or categories of problematic use (e.g., hazardous use vs. diagnosed disorder).

• Clarify whether pooled estimates will be stratified by:

Diagnostic vs. screening-based definitions, Past-year vs. lifetime prevalence.

• Consider whether combining these constructs risks inflating or obscuring clinically meaningful prevalence estimates.

Response: We thank the reviewer for pointing out the conceptual heterogeneity in how problematic substance use is defined, particularly with respect to differences in clinical severity (e.g., problematic use vs. diagnosable disorder) and time frame (e.g., past year vs. lifetime). We agree that pooling these constructs without distinction may obscure clinically meaningful differences.

In response (as well as other reviewer comments), we have refined our analytic framework to more clearly distinguish between levels of severity and assessment approaches. Specifically, Substance Use Disorder (SUD) status is now designated as the primary outcome, defined based on DSM or ICD diagnostic criteria. Problematic use, as identified by validated screening instruments (e.g., AUDIT, CUDIT), as the secondary outcome (see below for how this will be identified; see Table 1).

In terms of the time frame, where sufficient data are available, we will prioritize past year (or current) estimates for both SUD diagnoses and problematic use, given their greater clinical relevance and comparability across studies. Lifetime estimates will be analyzed separately and not pooled with past-year estimates. We have updated our planned stratification analyses in line with reviewer feedback (see pp. 10-11).

2. Decision to Average Multiple Prevalence Estimates Within Studies. The protocol states that when studies report multiple prevalence estimates for the same substance, a mean prevalence rate will be calculated. This approach is methodologically concerning, as it: Violates assumptions of independence, masks subgroup variability, may introduce bias depending on subgroup sizes.

Suggestions:

• Justify this approach more explicitly with citations from prevalence meta-analysis methodology.

• Consider alternative strategies, such as:

o Selecting the most clinically relevant estimate (e.g., total sample), conducting sensitivity analyses, using multilevel or robust variance meta-analytic approaches.

Response: We thank the reviewer for this helpful comment and suggestion. First, when studies provide a total sample estimate, we will extract that estimate (even if subgroups are also identified). When studies report multiple subgroup-specific prevalence estimates (e.g., by sex, age group, or different pain conditions), we will derive a single study-level prevalence by aggregating subgroup data using sample size-weighted estimates (i.e., total number of cases divided by the total sample size, where available; see pp. 10). This approach retains within-study information while minimizing bias associated with unweighted averaging of prevalence rate and ensures that each study contributes only one independent estimate per substance. To assess the robustness of this approach, following the reviewer’s recommendation, we will also conduct sensitivity analyses using alternative strategies, such as selecting the largest subgroup, as well as serially excluding each study to determine the influence of individual studies on the overall prevalence estimates. Although multilevel and robust variance estimation methods can account for dependent effect sizes, we will prioritize a single aggregated estimate per study to enhance interpretability and maintain comparability across studies, which is common practice in prevalence meta-analyses (see citations below for examples).

• Barendregt, J. J., Doi, S. A., Lee, Y. Y., Norman, R. E., & Vos, T. (2013). Meta-analysis of prevalence. J epidemiol community health, 67(11), 974-978.

• Rotenstein LS, Ramos MA, Torre M, et al. Prevalence of Depression, Depressive Symptoms, and Suicidal Ideation Among Medical Students: A Systematic Review and Meta-Analysis. JAMA. 2016;316(21):2214–2236. doi:10.1001/jama.2016.17324

• Borenstein, M., Hedges, L. V., Higgins, J. P., & Rothstein, H. R. (2010). A basic introduction to fixed‐effect and random‐effects models for meta‐analysis. Research synthesis methods, 1(2), 97-111.

• Sterne, J. A., Jüni, P., Schulz, K. F., Altman, D. G., Bartlett, C., & Egger, M. (2002). Statistical methods for assessing the influence of study characteristics on treatment effects in ‘meta‐epidemiological’research. Statistics in medicine, 21(11), 1513-1524.

• Van Houwelingen, H. C., Arends, L. R., & Stijnen, T. (2002). Advanced methods in meta‐analysis: multivariate approach and meta‐regression. Statistics in medicine, 21(4), 589-624.

3. Anticipated Heterogeneity and Its Implications

Given the wide range of: Chronic pain definitions, pain conditions, substance types, products, and routes of administration, measurement tools, very high heterogeneity is likely.

Suggestions:

• Elaborate on how extreme heterogeneity (e.g., I² > 90%) will be handled in interpretation.

• Clarify whether pooled estimates will still be presented under such conditions, or whether narrative synthesis thresholds will be applied.

• Consider pre-specifying criteria for when meta-analysis may be deemed inappropriate.

Response: We thank the reviewer for this important comment and agree that substantial heterogeneity is likely given the diversity of chronic pain populations, substances, and measurement approaches included in this review.

We have revised the protocol to clarify our approach to the assessment and interpretation of heterogeneity. Specifically, we now emphasize that I² will not be interpreted using rigid thresholds, as recent methodological work (Migliavaca et al., 2022) demonstrates that very high I² values (e.g., >90%) are common in prevalence meta-analyses and may not be discriminative of meaningful heterogeneity. Instead, we will interpret I² in conjunction with τ², prediction intervals, and the clinical and methodological comparability of included studies (Migliavaca et al., 2022; see pp. 11-12).

We further clarify that pooled estimates will be presented using random-effects models even in the presence of substantial heterogeneity but will be interpreted cautiously as average prevalence across diverse contexts rather than precise summary values. To enhance interpretability, we will report prediction intervals to reflect the expected range of true prevalence estimates across settings (see p. 12).

In addition, we have specified that subgroup and, where feasible, meta-regression analyses will be conducted based on a priori hypotheses to explore potential sources of heterogeneity. Importantly, we now explicitly state that decisions regarding the appropriateness of meta-analysis will not be based solely on I². If heterogeneity is judged to be too substantial to support a meaningful pooled estimate (e.g., due to extreme variability that cannot be explained or limited conceptual comparability across studies), we will supplement or replace pooled estimates with a narrative synthesis, focusing on the distribution and range of estimates across studies (see p. 12).

• Migliavaca, C.B., Stein, C., Colpani, V., Barker, T.H., Ziegelmann, P.K., Munn, Z., & Falavigna, M. (2022). Meta-analysis of prevalence: I² statistic and how to deal with heterogeneity. Research Synthesis Methods, 13(3), 363–367. https://doi.org/10.1002/jrsm.1547

4. Exclusion of Non-English Full-Text Articles: While resource constraints are understandable, excluding non-English full texts may bias prevalence estimates, particularly given global variation in chronic pain and substance use.

Suggestions:

• Explicitly acknowledge this as a source of language and regional bias in both the Methods and Discussion.

• Clarify whether non-English abstracts will contribute to any descriptive mapping of excluded studies (beyond listing in an appendix).

Response: We have explicitly acknowledged that excluding non-English full texts may be a source of language and regional bias (see p. 8). For all identified non-English papers, we will 1) indicate in the PRISMA flow diagram under a dedicated exclusion category of "unable to obtain full translation," distinct from other exclusion reasons, to allow readers to gauge the potential scope of this bias, and 2) provide a summary table in the appendix that includes, where extractable from the abstract alone, the study country, pain condition, substance type examined, and reported prevalence if clearly stated in the abstract. Importantly, if there is a clear pattern that is identified from these non-English articles, we will note this in text.

5. Co-use of Substances

The protocol states that studies reporting co-use will be included, but the analytic handling of co-use is unclear.

Suggestions:

• Clarify whether co-use prevalence will be: Reported descriptively, analyzed separately, or excluded from pooled estimates.

• Explicitly state whether individuals may be counted multiple times across substance-specific analyses.

Response: We agree that the handling of co-use requires clarification. We have revised the protocol (see p. 9) to specify that prevalence estimates of co-use (i.e., concurrent problematic use or use disorders involving multiple substances) will be extracted and reported descriptively. Where sufficient data are available, co-use will also be examined in exploratory analyses; however, co-use estimates will not be included in the primary pooled prevalence analyses for individual substances.

Our primary analyses focus on estimating substance-specific prevalence (i.e., use disorders for nicotine, alcohol, and cannabis separately) and secondary analyses for problematic use. Accordingly, each study will contribute at most one prevalence estimate per substance to the pooled analyses. For example, if a study reports that 30% of participants meet criteria for alcohol use disorder, 40% for nicotine dependence, and 15% for co-occurring alcohol and nicotine use disorder, the alcohol (30%) and nicotine (40%) estimates would be included in their respective meta-analyses, while the co-use estimate (15%) would be summarized descriptively or examined in secondary analyses.

Because the analyses focus on substance-specific prevalence, each substance will be examined separately. As a result, individuals who use multiple substances may contribute to more than one substance-specific analysis (e.g., both alcohol and nicotine). However, within each substance-specific meta-analysis, each study will contribute only one estimate, and individuals will not be counted more than once. We will also descriptively report how co-use was defined in each of the estimates used. We have clarified this approach on pp. 10-11 to improve transparency.

Minor Comments

6. Terminology consistency

The manuscript alternates between “problematic use,” “misuse,” “hazardous use,” and “use disorder.”

Recommend standardizing terminology and providing a brief definitional table.

Response: We have added a table to help with clarity and standardization of terminology (see Table 2), and have clarified that the primary outcomes now focus on use disorders, with secondary outcomes focused on problematic use.

7. Grammar and clarity

Minor typographical issues (e.g., duplicated phrases such as “full the full searches”) should be corrected.

Some long sentences in the Introduction could be tightened for clarity.

Response: We have thoroughly reviewed the manuscript for typographical and grammar errors (see revised manuscript).

8. Data Availability Statement

While acceptable for a protocol, the statement could be strengthened by briefly outlining where and how extracted data and analytic code will be shared upon completion (e.g., OSF, GitHub).

Response: We have added additional details on where the data and code will be shared (see p. Data Availability Statement). Specifically, the extracted data and analytic code will be shared on OSF upon completion.

9. Table 1

Consider explicitly stating whether mixed pain populations (e.g., cancer + non-cancer pain) are excluded or handled separately.

Response: We have revised the protocol to specify that studies including mixed pain populations (e.g., cancer and non-cancer pain) will be included only if data specific to individuals with chronic non-cancer pain can be extracted separately (see Table 1 note).

Reviewer #2

Overall Recommendation

This is a well-motivated and potentially impactful protocol, addressing an important and understudied area at the intersection of chronic pain and non-opioid substance use. With clearer conceptual definitions, stronger justification for analytic decisions, and improved handling of heterogeneity, the manuscript would meet high methodological standards and provide a solid foundation for the planned review.

Recommendation: Major revision

This protocol addresses a critical and timely gap in the literature regarding the prevalence of problematic nicotine, alcohol, and cannabis use among adults with chronic pain. While opioid use has been extensively characterized, the scope of non-opioid substance use in this population remains less precisely defined. The protocol is methodologically strong overall, adheres to PRISMA-P guidance, and outlines a thoughtful statistical plan. The study aims are well aligned with the stated need to better quantify prevalence estimates in this area. That said, some technical aspects related to the search strategy and data synthesis would benefit from additional clarification to ensure the highest standards of transparency and reproducibility.

1. Search Strategy Refinement – Seed Article Omission

The protocol notes that one seed article was not retrieved because it lacked “misuse” terminology. This raises the possibility that the current search string may be overly restrictive. It would be helpful to clarify whether the search strategy was subsequently modified to incorporate broader terminology (e.g., high-risk use, heavy use, or consumption-level descriptors), or whether the decision reflects an intentional prioritization of specificity over sensitivity.

Response: Thank you for this helpful comment. As noted, one seed article was not retrieved because the paper did not include any misuse-related terms in the title, abstract, or indexed subject headings. Following this observation, we reviewed and refined the search strategy to ensure broader coverage of relevant terminology. Specifically, we incorporated a comprehensive set of synonyms reflecting problematic use, including terms related to abuse, addiction, dependence, and overconsumption, as well as high-risk patterns of use (e.g., binge, daily, harmful, heavy, problematic, and risky use). We also include terms for alcoholism and specific database headings for alcohol and tobacco use disorders and marijuana abuse. Yet, the specific seed article was still not identified (see pp. 7-8).

2. Database Translation and Language Bias

The protocol indicates that articles may be excluded if translation is not feasible due to funding limitations. This introduces the potential for language and geographic bias. It would strengthen the protocol to explicitly state how this limitation will be rep

# Peer review: Decision Letter 1

**DOI:** 10.1371/journal.pone.0356365.r003

**Authors:** Marianna Mazza

27 May 2026

Dear Dr. Rogers,

Thank you for submitting your manuscript to PLOS ONE. After careful consideration, we feel that it has merit but does not fully meet PLOS ONE’s publication criteria as it currently stands. Therefore, we invite you to submit a revised version of the manuscript that addresses the points raised during the review process. Please follow with particular attention reviewer's 1 requests.

Please submit your revised manuscript by Jul 11 2026 11:59PM. If you will need more time than this to complete your revisions, please reply to this message or contact the journal office at plosone@plos.org. When you're ready to submit your revision, log on to https://www.editorialmanager.com/pone/ and select the 'Submissions Needing Revision' folder to locate your manuscript file.

- A letter that responds to each point raised by the academic editor and reviewer(s). You should upload this letter as a separate file labeled 'Response to Reviewers'.
- A marked-up copy of your manuscript that highlights changes made to the original version. You should upload this as a separate file labeled 'Revised Manuscript with Track Changes'.
- An unmarked version of your revised paper without tracked changes. You should upload this as a separate file labeled 'Manuscript'.

If you would like to make changes to your financial disclosure, please include your updated statement in your cover letter. Guidelines for resubmitting your figure files are available below the reviewer comments at the end of this letter.

If applicable, we recommend that you deposit your laboratory protocols in protocols.io to enhance the reproducibility of your results. Protocols.io assigns your protocol its own identifier (DOI) so that it can be cited independently in the future. For instructions see: https://journals.plos.org/plosone/s/submission-guidelines#loc-laboratory-protocols. Additionally, PLOS ONE offers an option for publishing peer-reviewed Lab Protocol articles, which describe protocols hosted on protocols.io. Read more information on sharing protocols at https://plos.org/protocols?utm_medium=editorial-email&utm_source=authorletters&utm_campaign=protocols.

As the corresponding author, your ORCID iD is verified in the submission system and will appear in the published article. PLOS supports the use of ORCID, and we encourage all coauthors to register for an ORCID iD and use it as well. Please encourage your coauthors to verify their ORCID iD within the submission system before final acceptance, as unverified ORCID iDs will not appear in the published article. *Only*  the individual author can complete the verification step; PLOS staff *cannot*  verify ORCID iDs on behalf of authors.

We look forward to receiving your revised manuscript.

Kind regards,

Marianna Mazza

Academic Editor

PLOS One

Journal Requirements:

If the reviewer comments include a recommendation to cite specific previously published works, please review and evaluate these publications to determine whether they are relevant and should be cited. There is no requirement to cite these works unless the editor has indicated otherwise.

[Note: HTML markup is below. Please do not edit.]

Reviewers' comments:

Reviewer's Responses to Questions

**Comments to the Author**

1. Does the manuscript provide a valid rationale for the proposed study, with clearly identified and justified research questions?

Reviewer #1: Yes

Reviewer #2: Yes

Reviewer #3: Yes

**********

2. Is the protocol technically sound and planned in a manner that will lead to a meaningful outcome and allow testing the stated hypotheses?

Reviewer #1: Partly

Reviewer #2: Yes

Reviewer #3: Yes

**********

3. Is the methodology feasible and described in sufficient detail to allow the work to be replicable?

Reviewer #1: No

Reviewer #2: Yes

Reviewer #3: Yes

**********

4. Have the authors described where all data underlying the findings will be made available when the study is complete?

The PLOS Data policy

Reviewer #1: No

Reviewer #2: Yes

Reviewer #3: Yes

**********

5. Is the manuscript presented in an intelligible fashion and written in standard English?

Reviewer #1: No

Reviewer #2: Yes

Reviewer #3: Yes

**********

Please use the space provided to explain your answers to the questions above and, if applicable, provide comments about issues authors must address before this protocol can be accepted for publication. You may also include additional comments for the author, including concerns about research or publication ethics.

You may also provide optional suggestions and comments to authors that they might find helpful in planning their study.

Reviewer #1: Major Comments

Persistent conceptual heterogeneity in “problematic use”

Although the revised protocol distinguishes substance use disorders from problematic use, the operationalization of “problematic use” remains broad and somewhat conceptually unstable. Terms such as “hazardous,” “risky,” “harmful,” “misuse,” and “unhealthy use” are grouped together, yet these constructs differ clinically, behaviorally, and diagnostically. Table 2 improves transparency but may still permit substantial conceptual heterogeneity.

Consider:

Explicitly prioritizing validated screening tools with established cutoffs,

Clarifying whether studies using author-defined thresholds without validation will be excluded,

Providing a more hierarchical framework for categorizing severity.

Pooling prevalence across highly heterogeneous populations

The planned pooling strategy may still yield prevalence estimates of questionable interpretability due to anticipated extreme heterogeneity across:

Pain conditions,

Clinical versus community settings,

Substance definitions,

Measurement tools,

Geographic contexts,

Timeframes,

Study designs.

Although the manuscript appropriately discusses heterogeneity and prediction intervals, the protocol should more explicitly define:

What level of conceptual heterogeneity would preclude meta-analysis,

Whether narrative synthesis would replace pooling under certain circumstances,

How interpretability of pooled prevalence will be communicated to readers when heterogeneity remains extreme despite subgroup analyses.

Inclusion of interventional studies requires stronger justification

The inclusion of randomized and non-randomized trials remains somewhat problematic for a prevalence-focused review. Even if only baseline data are extracted, trial populations are often highly selected and may not represent underlying chronic pain populations. This could introduce selection bias and inflate prevalence estimates.

The authors should:

More clearly justify inclusion of clinical trial samples,

Consider conducting separate subgroup analyses by study design,

Discuss the potential effect of enriched clinical samples on pooled prevalence estimates.

Potential duplication of populations

The strategy for identifying duplicate samples is appreciated but may still be insufficient. Reliance on author names, sample sizes, and study years may fail to identify overlapping cohorts from large longitudinal databases or multicenter studies.

The protocol would benefit from:

More explicit criteria for identifying overlapping samples,

Clarification on how partially overlapping datasets will be handled,

Whether preference will be given to larger or more comprehensive datasets.

Language bias remains a significant limitation

The revised discussion acknowledges exclusion of non-English full texts; however, the practical implications may be larger than described given global variation in chronic pain and substance use epidemiology.

The authors should discuss:

Whether machine translation tools (e.g., DeepL or Google Translate) will be used for feasibility screening,

Potential underrepresentation of studies from low- and middle-income countries,

How this may affect generalizability of prevalence estimates.

Potential confounding by medical cannabis legalization contexts

Cannabis use prevalence may vary dramatically across regions depending on legal status, medical prescribing frameworks, and recreational availability. This is particularly important because cannabis use in chronic pain populations may reflect therapeutic use rather than problematic use in some jurisdictions.

Consider including:

Legal status or region-specific cannabis policy as a moderator,

Distinction between prescribed medical cannabis and non-medical use where possible.

Risk-of-bias integration could be stronger

The addition of sensitivity analyses using risk-of-bias scores is valuable. However, reliance on JBI sum scores may oversimplify methodological quality. Some items may contribute differently to prevalence bias.

Consider:

Presenting domain-level assessments,

Exploring whether high-risk studies disproportionately influence pooled estimates,

Clarifying how disagreements in quality ratings will be resolved.

Minor Comments

The abstract is generally clear, but the sentence:

“Therefore, a systematic review and meta-analysis, characterizing the prevalence rates… are urgently needed”

should be grammatically revised for clarity.

Several sentences in the introduction are lengthy and could be condensed for readability, particularly in the sections discussing theoretical models and chronic overlapping pain conditions.

The protocol should clarify whether nicotine includes:

combustible tobacco,

vaping/e-cigarettes,

smokeless tobacco,

mixed nicotine products.

Similarly, cannabis terminology should clarify whether synthetic cannabinoids are included or excluded.

The manuscript alternates between “use disorders,” “dependence,” and “problematic use.” While improved from the prior version, terminology consistency could still be enhanced throughout the text.

Consider adding a schematic figure summarizing:

eligibility criteria,

outcome hierarchy,

analytic workflow,

subgroup analyses.

This would improve protocol clarity for readers.

The rationale for selecting the Sidik–Jonkman estimator over alternative estimators (e.g., REML or DerSimonian-Laird) could be briefly justified.

The manuscript should specify whether prevalence estimates will undergo variance-stabilizing transformation (e.g., Freeman–Tukey double arcsine transformation), which is commonly used in prevalence meta-analyses.

The statement:

“individuals will only be counted in one estimate and not be counted more than once”

is somewhat unclear because individuals may contribute to multiple substance-specific analyses. This wording should be revised for precision.

The discussion appropriately outlines implications for clinicians and policymakers; however, it may overstate the certainty of pooled estimates given anticipated heterogeneity. A slightly more cautious tone is recommended

Reviewer #2: The authors responded appropriately to the comments.

Reviewer #3: The authors have satisfactorily addressed all the previous queries. But I have one objection. Authors have mentioned, if prevalence data is available from the baseline from cohort or experimental study, then these studies will also be taken into consideration. But for the baseline information, how the methodological rigour of those studies will be judged? Moreover, in experimental studies, one group will be having intervention having the disease and control group will be having placebo having the disease. Then how prevalence will be estimated from experimental studies? Moreover, if baseline findings are taken, then again the same problem of judging the article in methodological rigour in addition to non-availability of some basic findings that are need for SRMA.

**********

what does this mean?). If published, this will include your full peer review and any attached files.

If you choose “no”, your identity will remain anonymous but your review may still be made public.

**Do you want your identity to be public for this peer review?**  For information about this choice, including consent withdrawal, please see our Privacy Policy

Reviewer #1: No

Reviewer #2: No

Reviewer #3: **Yes:** Dr. Indranil Saha

**********

[NOTE: If reviewer comments were submitted as an attachment file, they will be attached to this email and accessible via the submission site. Please log into your account, locate the manuscript record, and check for the action link "View Attachments". If this link does not appear, there are no attachment files.]

To ensure your figures meet our technical requirements, please review our figure guidelines: https://journals.plos.org/plosone/s/figures

You may also use PLOS’s free figure tool, NAAS, to help you prepare publication quality figures: https://journals.plos.org/plosone/s/figures#loc-tools-for-figure-preparation.

NAAS will assess whether your figures meet our technical requirements by comparing each figure against our figure specifications.

# Author comment: Author response to Decision Letter 2

**DOI:** 10.1371/journal.pone.0356365.r004

31 Jul 2026

Response Letter to Reviewers

We appreciate the thoughtful review and the opportunity to revise and resubmit this manuscript. We believe the suggested revisions have significantly strengthened the manuscript. In this letter, we outline how we addressed the feedback provided by reviewers. For ease of review, we have highlighted the corresponding changes in the manuscript in yellow.

Reviewer #1: Major Comments

Persistent conceptual heterogeneity in “problematic use”

Although the revised protocol distinguishes substance use disorders from problematic use, the operationalization of “problematic use” remains broad and somewhat conceptually unstable. Terms such as “hazardous,” “risky,” “harmful,” “misuse,” and “unhealthy use” are grouped together, yet these constructs differ clinically, behaviorally, and diagnostically. Table 2 improves transparency but may still permit substantial conceptual heterogeneity.

Consider:

Explicitly prioritizing validated screening tools with established cutoffs,

Clarifying whether studies using author-defined thresholds without validation will be excluded,

Providing a more hierarchical framework for categorizing severity.

Pooling prevalence across highly heterogeneous populations

Response: We thank the reviewer for their thoughtful comment. We agree that the operationalization of problematic use is broad and encompasses a heterogeneous group of constructs (e.g., hazardous, risky, harmful use, and misuse), and this variability reflects an important limitation of the existing literature. As noted in our revised protocol (see p. 6), the primary outcome of this review is now the prevalence of nicotine, alcohol, and cannabis use disorders, while problematic use is examined as a secondary outcome. This change was made specifically to distinguish clinically diagnosed substance use disorders from broader screening-based indicators of problematic use.

For the secondary analyses of problematic use, we have further clarified our approach following the reviewer’s suggestion. Specifically, we will include only prevalence estimates derived from validated screening instruments using established cutoffs (e.g., AUDIT, CUDIT, FTND). Studies relying solely on author-defined thresholds without evidence of validation will not contribute to the pooled prevalence estimates of problematic use (see p. 9). During data extraction, we will also document the specific construct assessed (e.g., hazardous use, harmful use, misuse) and the corresponding instrument used. Where sufficient data are available, we will conduct subgroup or moderator analyses by screening instrument or measure type to examine whether prevalence estimates differ across these measures (see p. 12). These revisions are intended to improve transparency while acknowledging that some conceptual heterogeneity will inevitably remain because of differences in how problematic use has been operationalized across the published literature.

The planned pooling strategy may still yield prevalence estimates of questionable interpretability due to anticipated extreme heterogeneity across:

Pain conditions,

Clinical versus community settings,

Substance definitions,

Measurement tools,

Geographic contexts,

Timeframes,

Study designs.

Although the manuscript appropriately discusses heterogeneity and prediction intervals, the protocol should more explicitly define:

What level of conceptual heterogeneity would preclude meta-analysis,

Whether narrative synthesis would replace pooling under certain circumstances,

How interpretability of pooled prevalence will be communicated to readers when heterogeneity remains extreme despite subgroup analyses.

Response: We thank the reviewer for this thoughtful comment and agree that conceptual heterogeneity is an important consideration in prevalence meta-analyses.

First, regarding what level of conceptual heterogeneity would preclude meta-analysis, there is no universally accepted threshold for conceptual heterogeneity. Accordingly, we elected not to rely on a rigid criterion. Instead, quantitative synthesis will be undertaken only when studies are judged to be sufficiently comparable with respect to key conceptual characteristics, including the chronic pain population, substance definitions, measurement approach, and study designs. This approach is consistent with guidelines that suggest that studies should be grouped for meta-analytic synthesis based on pre-defined conceptual comparable characteristics and not use meta-analysis as a default when summary estimates would be misleading.

Lin, L. (2020). Comparison of four heterogeneity measures for meta‐analysis. Journal of Evaluation in Clinical Practice, 26(1), 376-384.

Melsen, W. G., Bootsma, M. C. J., Rovers, M. M., & Bonten, M. J. M. (2014). The effects of clinical and statistical heterogeneity on the predictive values of results from meta-analyses. Clinical Microbiology and Infection, 20(2), 123-129.

Campbell, M., McKenzie, J. E., Sowden, A., Katikireddi, S. V., Brennan, S. E., Ellis, S., ... & Thomson, H. (2020). Synthesis without meta-analysis (SWiM) in systematic reviews: Reporting guideline. BMJ, 368.

Second, if the included studies are judged to be too conceptually heterogenous for a pooled prevalence estimate to provide a meaningful summary of the evidence (determined by expert consensus from the study team), we will instead provide a narrative synthesis with additional transparent reporting and visual presentation and range of prevalence rates across studies.

Third, we clarified that, when meta-analysis is conducted despite substantial heterogeneity, prevalence estimates will be interpreted as descriptive summaries rather than single generalizable estimates. We will emphasize prediction intervals, subgroup patterns, and the limits of generalizability in the Results and Discussion (see p. 12).

Inclusion of interventional studies requires stronger justification

The inclusion of randomized and non-randomized trials remains somewhat problematic for a prevalence-focused review. Even if only baseline data are extracted, trial populations are often highly selected and may not represent underlying chronic pain populations. This could introduce selection bias and inflate prevalence estimates.

The authors should:

More clearly justify inclusion of clinical trial samples,

Consider conducting separate subgroup analyses by study design,

Discuss the potential effect of enriched clinical samples on pooled prevalence estimates.

Response: We agree that participants enrolled in randomized and non-randomized trials may differ from the broader population of individuals with chronic pain because of trial-specific eligibility criteria and treatment-seeking status. However, we believe these studies remain informative for the current review because many report baseline prevalence estimates that reflect clinically relevant chronic pain populations represented in the published literature.

To address this concern, we have further clarified that these interventional studies will contribute only baseline (pre-intervention) prevalence data (see p. 7). Mid- and post-intervention, as well as follow-up data will NOT be included in the prevalence analyses. These data will be treated as observational prevalence estimates and will not be combined with any post-treatment data. We will also conduct separate subgroup analyses by study design whenever sufficient studies are available (see pp. 11-12). This will allow us to evaluate whether prevalence estimates differ systematically between cross-sectional, cohort, and interventional studies. In addition, we will discuss the findings from these subgroup analysis, as well as the potential impact of including these clinical trial samples on pooled prevalence estimates and their generalizability, in the Discussion.

Potential duplication of populations

The strategy for identifying duplicate samples is appreciated but may still be insufficient. Reliance on author names, sample sizes, and study years may fail to identify overlapping cohorts from large longitudinal databases or multicenter studies.

The protocol would benefit from:

More explicit criteria for identifying overlapping samples,

Clarification on how partially overlapping datasets will be handled,

Whether preference will be given to larger or more comprehensive datasets.

Response: We agree that identifying overlapping study populations can be challenging, particularly for large publicly available longitudinal cohorts and multicenter studies. We have therefore expanded our protocol to further clarify how potential duplicate samples will be identified and handled. Specifically, in addition to study authors, sample size, publication year, and study measures, we will also examine the cohort or database name (when applicable), recruitment period, and other relevant study characteristics (e.g., geographical locations) to identify potentially overlapping samples (see p. 11).

When multiple publications report the same prevalence outcome from overlapping samples, only one independent estimate will be included in the corresponding meta-analysis. Preference will be given to the report with the largest eligible sample and/or the most comprehensive prevalence data relevant to the planned analysis. We have clarified these procedures in the Methods (p. 11).

Language bias remains a significant limitation

The revised discussion acknowledges exclusion of non-English full texts; however, the practical implications may be larger than described given global variation in chronic pain and substance use epidemiology.

The authors should discuss:

Whether machine translation tools (e.g., DeepL or Google Translate) will be used for feasibility screening,

Potential underrepresentation of studies from low- and middle-income countries,

How this may affect generalizability of prevalence estimates.

Response: We appreciate the reviewer’s concerns regarding the exclusion of non-English full text articles. We have clarified that machine translation tools (e.g., Google Translate) will be used during feasibility screening to help determine whether non-English studies appear to meet the eligibility criteria and whether sufficient information can be extracted (see p. 8). However, because formal translation of full-text articles is beyond the scope of the current review, studies that cannot be adequately translated will be excluded. We have also expanded the Discussion to acknowledge that there is potential underrepresentation of prevalence estimates from low- and middle-income countries, where publication in languages other than English may be more common. Consequently, the pooled prevalence estimates may not fully reflect the global epidemiology of substance use disorders among individuals with chronic pain (see p. 14).

Potential confounding by medical cannabis legalization contexts

Cannabis use prevalence may vary dramatically across regions depending on legal status, medical prescribing frameworks, and recreational availability. This is particularly important because cannabis use in chronic pain populations may reflect therapeutic use rather than problematic use in some jurisdictions.

Consider including:

Legal status or region-specific cannabis policy as a moderator,

Distinction between prescribed medical cannabis and non-medical use where possible.

Response: Our protocol already included planned subgroup analyses comparing medical versus non-medical cannabis use where sufficient data are available (see p. 12). To further address the reviewer's concern, we have additionally included jurisdiction-specific cannabis legal status as a planned moderator, where sufficient information is available, and clarified these analyses in the Methods (p. 12).

Risk-of-bias integration could be stronger

The addition of sensitivity analyses using risk-of-bias scores is valuable. However, reliance on JBI sum scores may oversimplify methodological quality. Some items may contribute differently to prevalence bias.

Consider:

Presenting domain-level assessments,

Exploring whether high-risk studies disproportionately influence pooled estimates,

Clarifying how disagreements in quality ratings will be resolved.

Response: We thank the reviewer for this thoughtful comment. In response, we have clarified that the JBI Critical Appraisal Checklist will continue to be used because it is specifically designed and widely recommended for prevalence studies. However, risk of bias will not be interpreted solely on the basis of an overall summary score. In addition to conducting sensitivity analyses excluding studies judged to be at high risk of bias, we will report the individual JBI domain ratings for each included study in the supplementary materials to improve transparency. Finally, we have clarified that risk-of-bias assessments will be conducted independently by two reviewers, with any disagreements resolved through discussion and, when necessary, consultation with a third reviewer. We have revised the Methods accordingly (pp. 9-10).

Minor Comments

The abstract is generally clear, but the sentence:

“Therefore, a systematic review and meta-analysis, characterizing the prevalence rates… are urgently needed”

should be grammatically revised for clarity.

Response: We have revised the sentence accordingly in the Abstract.

Several sentences in the introduction are lengthy and could be condensed for readability, particularly in the sections discussing theoretical models and chronic overlapping pain conditions.

Response: We have reviewed and revised the Introduction section accordingly.

The protocol should clarify whether nicotine includes:

combustible tobacco,

vaping/e-cigarettes,

smokeless tobacco,

mixed nicotine products.

Similarly, cannabis terminology should clarify whether synthetic cannabinoids are included or excluded.

The manuscript alternates between “use disorders,” “dependence,” and “problematic use.” While improved from the prior version, terminology consistency could still be enhanced throughout the text.

Consider adding a schematic figure summarizing:

eligibility criteria,

outcome hierarchy,

analytic workflow,

subgroup analyses.

This would improve protocol clarity for readers.

Response: We appreciate the reviewer comments, and we have clarified the scope of nicotine and cannabis products included in the review. Specifically, nicotine-related outcomes may include combustible tobacco, electronic cigarettes/vaping products, smokeless tobacco, and other nicotine-containing products when assessed as part of nicotine use disorder or problematic nicotine use. Similarly, we have clarified the eligibility criteria for cannabis-related outcomes, including whether studies of synthetic cannabinoids will be eligible (see Table 1).

Following the previous revision, the protocol now clearly distinguishes substance use disorders as the primary outcome from problematic use as the secondary outcome. The term dependence is retained only when referring to historical diagnostic terminology (e.g., DSM-IV or ICD definitions) or the terminology used in the original studies. We are electing to keep this language to be consistent with clinically applicable terminology.

We appreciate the suggestion to include a schematic summarizing the study design and analytic workflow, and have included this to improve transparency (see Figure 1).

The rationale for selecting the Sidik–Jonkman estimator over alternative estimators (e.g., REML or DerSimonian-Laird) could be briefly justified.

Response: We thank the reviewer for this helpful suggestion. Upon further consideration, we agree that restricted maximum likelihood (REML) is a more appropriate choice for the planned analyses (see p. 12). Accordingly, we have revised the statistical analysis plan to use REML estimation rather than the previously proposed Sidik-Jonkman estimator. REML is a widely used estimator for random-effects meta-analysis and generally provides stable estimation of between-study variance, particularly when substantial heterogeneity is anticipated.

The manuscript should specify whether prevalence estimates will undergo variance-stabilizing transformation (e.g.

# Editor report: Decision Letter 2

**DOI:** 10.1371/journal.pone.0356365.r005

**Authors:** Marianna Mazza

3 Aug 2026

Prevalence of Nicotine, Alcohol, and Cannabis Use Disorders Among Adults with Chronic Pain: Protocol for a Systematic Review and Meta-Analysis

PONE-D-25-57476R2

Dear Dr. Rogers,

We’re pleased to inform you that your manuscript has been judged scientifically suitable for publication and will be formally accepted for publication once it meets all outstanding technical requirements.

Within one week, you’ll receive an e-mail detailing the required amendments. When these have been addressed, you’ll receive a formal acceptance letter and your manuscript will be scheduled for publication.

An invoice will be generated when your article is formally accepted. Please note, if your institution has a publishing partnership with PLOS and your article meets the relevant criteria, all or part of your publication costs will be covered. Please make sure your user information is up-to-date by logging into Editorial Manager at Editorial Manager® and clicking the ‘Update My Information' link at the top of the page. For questions related to billing, please contact billing support.

If your institution or institutions have a press office, please notify them about your upcoming paper to help maximize its impact. If they’ll be preparing press materials, please inform our press team as soon as possible -- no later than 48 hours after receiving the formal acceptance. Your manuscript will remain under strict press embargo until 2 pm Eastern Time on the date of publication. For more information, please contact onepress@plos.org.

Kind regards,

Marianna Mazza

Academic Editor

PLOS One

Additional Editor Comments (optional):

Reviewers' comments:

# Editor report: Acceptance letter

**DOI:** 10.1371/journal.pone.0356365.r006

**Authors:** Marianna Mazza

PONE-D-25-57476R2

PLOS One

Dear Dr. Rogers,

I'm pleased to inform you that your manuscript has been deemed suitable for publication in PLOS One. Congratulations! Your manuscript is now being handed over to our production team.

At this stage, our production department will prepare your paper for publication. This includes ensuring the following:

* All references, tables, and figures are properly cited

* All relevant supporting information is included in the manuscript submission,

* There are no issues that prevent the paper from being properly typeset

You will receive further instructions from the production team, including instructions on how to review your proof when it is ready. Please keep in mind that we are working through a large volume of accepted articles, so please give us a few days to review your paper and let you know the next and final steps.

Lastly, if your institution or institutions have a press office, please let them know about your upcoming paper now to help maximize its impact. If they'll be preparing press materials, please inform our press team within the next 48 hours. Your manuscript will remain under strict press embargo until 2 pm Eastern Time on the date of publication. For more information, please contact onepress@plos.org.

You will receive an invoice from PLOS for your publication fee after your manuscript has reached the completed accept phase. If you receive an email requesting payment before acceptance or for any other service, this may be a phishing scheme. Learn how to identify phishing emails and protect your accounts at https://explore.plos.org/phishing.

If we can help with anything else, please email us at customercare@plos.org.

Thank you for submitting your work to PLOS One and supporting open access.

Kind regards,

PLOS ONE Editorial Office Staff

on behalf of

Dr. Marianna Mazza

Academic Editor

PLOS One
